# Elevated anti-apoptotic shift in primary human aniridia limbal stromal cells following 48 hours supraphysiological glucose exposure, *in vitro*

**DOI:** 10.1371/journal.pone.0340117

**Published:** 2026-01-12

**Authors:** Shanhe Liu, Shuailin Li, Shao-Lun Hsu, Fabian N. Fries, Zhen Li, Swarnali Kundu, Berthold Seitz, Maryam Amini, Shweta Suiwal, Tanja Stachon, Nóra Szentmáry

**Affiliations:** 1 Dr. Rolf M. Schwiete Center for Limbal Stem Cell and Congenital Aniridia Research, Saarland University, Homburg, Saarland, Germany; 2 Department of Experimental Ophthalmology, Saarland University, Homburg, Saarland, Germany; 3 Department of Ophthalmology, Saarland University Medical Center, Homburg, Saarland, Germany; Huntsman Cancer Institute Cancer Hospital: University of Utah Health Huntsman Cancer Institute, UNITED STATES OF AMERICA

## Abstract

**Purpose:**

Previous work demonstrated that supraphysiological glucose remodels TGF-β1 and NF-κB signaling in human limbal stromal cells (LSCs) and congenital aniridia-derived LSCs (AN-LSCs). The present study investigated whether the same metabolic stress also alters apoptotic pathways in these cells.

**Methods:**

Primary LSCs (n = 12) and AN-LSCs (n = 8) were cultured for 48 hours in DMEM containing either normal (17.5 mM) or high (70 mM) glucose. Apoptosis was quantified by Annexin V/propidium iodide (PI) flow cytometry (FC). Expression levels of apoptosis-related genes—including *CASP3/7/8/9/10, BCL2, BID, BAX, CDKN1A (p21), CDKN1B (p27), TNFα, XIAP,* and *BIRC5* (Survivin)—were assessed by qPCR. Protein levels of these markers were analyzed by FC, and TNFα protein concentrations in culture supernatants were measured by ELISA.

**Results:**

High glucose significantly reduced the proportion of apoptotic cells in both LSCs (p = 0.0170) and AN-LSCs (p = 0.0181). In both cell types, *CASP8* (p = 0.0448; p = 0.0171) and *CASP10* (p = 0.0001; p = 0.0007) mRNA levels decreased, while *XIAP* (p = 0.0375; p = 0.0442) and *BIRC5* (p = 0.0196; p = 0.0003) were upregulated. AN-LSCs additionally showed reductions in *CASP3* (p = 0.0138) and *CDKN1A* (p = 0.0331), and exhibited lower *BAX* levels than LSCs under high glucose (p = 0.0255). Protein analysis corroborated these findings in AN-LSCs: Caspase-3 (p = 0.0154) and Caspase-8 (p = 0.0257) decreased, while Bcl-2 (p = 0.0284) and Survivin (p = 0.0467) levels increased. XIAP protein levels rose in both LSCs (p = 0.0451) and AN-LSCs (p = 0.0134).

**Conclusions:**

A 48-hour exposure to 70 mM glucose induces a marked anti-apoptotic shift in human limbal stromal cells, more pronounced in cells derived from congenital aniridia patients. Together with previous evidence on TGF-β1 and NF-κB regulation, these findings suggest that limbal cells may mount an early protective response to metabolic stress, which could be harnessed therapeutically to manage aniridia-associated keratopathy through coordinated survival and stress pathways.

## Introduction

Congenital aniridia is a rare panocular disorder caused primarily by heterozygous mutations in the PAX6 gene In addition to the hallmark feature of iris hypoplasia, patients with aniridia frequently exhibit a broad spectrum of ocular abnormalities, including cataract, glaucoma, foveal hypoplasia, and notably, a progressive corneal opacification known as aniridia-associated keratopathy (AAK) [1,2]. AAK develops in the majority of aniridia cases and typically results in a gradual deterioration of vision over time [3]. The principal driver of AAK is a progressive limbal stem cell deficiency (LSCD), characterized by the failure of the limbal epithelial stem cell niche, which leads to conjunctivalization and vascularization of the cornea [4–6]. However, the pathogenesis of AAK is multifactorial. Contributing factors include impaired corneal wound healing due to abnormal extracellular matrix turnover, a fragile corneal epithelium with defective differentiation, and reduced expression of adhesion molecules, all of which have been linked to PAX6 haploinsufficiency [7].

While AAK has traditionally been attributed to the loss of limbal epithelial stem cells, emerging evidence indicates that limbal stromal cells (LSCs) also play a critical role in disease progression [8,9]. LSCs reside within the corneal limbal stroma and support the epithelial stem cell niche; they are capable of modulating wound healing, fibrosis, and inflammation in the cornea [10]. Recent studies have demonstrated that LSCs derived from aniridia patients (AN-LSCs) differ fundamentally from healthy LSCs. For example, AN-LSCs exhibit significantly reduced *PAX6* mRNA levels and display altered levels of keratocyte differentiation markers—such as collagens, aldehyde dehydrogenase 3 family members, and keratocan—compared to controls [11]. These differences may underlie a predisposition to aberrant corneal matrix remodeling and the progression of AAK. Furthermore, AN-LSCs appear to possess an intrinsically altered stress response profile: baseline NF-κB protein levels are reported to be lower in AN-LSCs than in healthy LSCs. Given that NF-κB is a key transcription factor regulating inflammation and cell survival, its reduced basal activity suggests a distinct signaling environment in the aniridic limbus [12]. Consistently, our group and others have observed that limbal stromal cells from aniridia patients exhibit heightened sensitivity to various stressors and pharmacologic agents [12–15]. Collectively, these findings indicate that, beyond epithelial stem cell loss, stromal cell dysfunction represents a crucial component of AAK pathophysiology.

Metabolic stress is a key factor that can profoundly affect ocular cell survival and function. In our previous work, we demonstrated that supraphysiological glucose concentrations (70 mM) significantly remodel TGF-β1 and NF-κB signaling in both human limbal stromal cells (LSCs) and congenital aniridia–derived LSCs (AN-LSCs), *in vitro*. Specifically, high glucose initially activated antioxidant defense mechanisms, evidenced by upregulation of Nrf2 and catalase, while concurrently downregulating key regulatory genes such as TGF-β1, SMAD2/3, and NF-κB—effects that were particularly pronounced in AN-LSCs. These alterations were time-dependent: early adaptive responses were followed by partial rebound or exhaustion of these signaling pathways with prolonged exposure. This prior study highlighted a compromised stress-adaptive signaling profile in AN-LSCs, suggesting an increased susceptibility to metabolic perturbations [8]. In addition, elevated glucose levels—such as those present in diabetes mellitus—have detrimental effects on the cornea and limbus. Diabetic patients frequently experience delayed corneal wound healing and neuropathy, partly due to hyperglycemia-induced biochemical disruptions [16]. A previous study reported a 5.6% incidence of diabetes mellitus among subjects with congenital aniridia [17]. Hyperglycemia can disturb the balance of growth factors like TGF-β in the cornea; for instance, it has been shown that high glucose suppresses TGF-β3 while sustaining TGF-β1, thereby delaying corneal epithelial wound closure in diabetic mice [18]. At the cellular level, high glucose induces oxidative and nitrosative stress, leading to the accumulation of reactive oxygen and nitrogen species such as superoxide, nitric oxide, and peroxynitrite [19]. These reactive species can damage cellular components and activate stress signaling pathways. Numerous studies using diabetes models have demonstrated that hyperglycemia triggers apoptosis in various ocular cell types. High glucose can activate multiple pro-apoptotic proteins, including caspases and Bcl-2 family members, ultimately culminating in programmed cell death. Such glucose-induced apoptosis has been implicated in diabetic complications affecting tissues from the retina to the corneal endothelium [19–21]. Conversely, cells under metabolic stress may also engage protective mechanisms; for example, transient high glucose exposure can activate antioxidant defenses, such as the Nrf2 pathway, to counteract oxidative stress [8]. Thus, the net effect of acute high glucose on cell fate likely depends on both the duration and the cellular context of exposure.

In the context of the limbus, there is growing interest in how metabolic stress might influence limbal cell populations and, by extension, corneal health. We recently reported that exposure to supraphysiological glucose concentrations (70 mM) profoundly alters key signaling pathways in primary limbal fibroblasts. After 48 hours of high-glucose exposure, TGF-β1 expression was significantly reduced in both healthy LSCs and AN-LSCs, while transcripts of antioxidant enzymes (such as Nrf2 and catalase) were upregulated. Notably, NF-κB expression also decreased under high glucose, particularly in aniridic cells, accompanied by reductions in hypoxia-inducible factor 1α (HIF-1α).

These acute responses suggest that metabolic stress initially suppresses fibrogenic and inflammatory signaling, potentially as an adaptive mechanism. However, with prolonged exposure (72 hours), we observed a rebound increase in *TGF-β1* mRNA levels and a rise in *NF-κB* expression (at the mRNA level in healthy LSCs), indicating a complex, time-dependent remodeling of these signaling pathways. The perturbation of the TGF-β1 and NF-κB pathways by high glucose underscores that limbal stromal cells mount a significant stress response, characterized by both protective (antioxidant) and potentially pathogenic (fibrotic and inflammatory) changes. Given that TGF-β1 and NF-κB are also key modulators of apoptosis and cell survival, these findings prompted us to investigate whether the apoptotic machinery of limbal cells is similarly affected by hyperglycemic stress [8].

Apoptosis plays a critical role in maintaining tissue homeostasis, and dysregulation of apoptotic pathways can significantly impact corneal clarity and wound healing [22,23]. Inflammatory cytokines present in AAK, such as TNF-α, may trigger apoptotic cascades in limbal cells, potentially worsening LSCD by depleting the residual cell population [12]. Conversely, an inherent or adaptive anti-apoptotic bias in limbal cells could be protective, preserving the stem cell niche under stress. However, whether elevated glucose conditions influence apoptotic signaling in limbal stromal cells had not yet been investigated.

Given previous evidence that AN-LSCs display altered baseline and stress-induced signaling in key survival and inflammatory pathways, we analyzed healthy LSCs and AN-LSCs in parallel to determine whether high-glucose exposure elicits a general early stress adaptation in limbal stromal cells, or a response that is amplified or remodeled within the PAX6-haploinsufficient context. We hypothesized that high-glucose exposure would modulate the expression of key apoptotic regulators—including caspases, BCL-2 family proteins, and inhibitor of apoptosis proteins—in LSCs, and that AN-LSCs might display a distinct apoptotic response due to their PAX6-related abnormalities. To test this, the present study examined primary limbal stromal cells from healthy donors and congenital aniridia patients *in vitro*, comparing their apoptotic profiles under physiological versus high-glucose conditions. We quantified apoptosis rates and assessed gene and protein expression levels of major apoptosis-related molecules. By linking these findings to our previous observations on TGF-β1 and NF-κB signaling, we aim to provide a comprehensive understanding of how metabolic stress governs survival and death decisions in limbal stromal cells. This knowledge may ultimately support the development of future AAK therapies that target coordinated cell survival and stress response pathways.

## Materials and methods

### Ethics statement

All procedures were conducted in accordance with the tenets of the Declaration of Helsinki and were approved by the Saarland Ethics Committee, Germany (approval no. 178/22). Written informed consent was obtained from all participants. The prospective study enrolled patients between 7 November 2022 and 15 December 2024.

### Human limbal tissue procurement

Human limbal biopsies (1.5 mm in diameter) were obtained during clinically indicated surgeries from eight eyes of patients with congenital aniridia treated at the Department of Ophthalmology, Saarland University Medical Center (mean ± SD age: 32.5 ± 17.1 years; range: 2–50 years; 37.5% male) (Table 1). The severity of AAK was graded by slit-lamp biomicroscopy according to the criteria of Lagali et al. [9,24]. Control biopsies were collected from twelve healthy donor eyes provided by the LIONS Cornea Bank Saar-Lor-Lux, Trier/Westpfalz (mean age: 73.5 ± 15.5 years; range: 58–89 years; 37.5% male) (Table 2).

**Table 1:** Detailed information on the included subjects with congenital aniridia is provided. The table includes the aniridia-associated keratopathy (AAK) grade according to Lagali et al. [11 25], as well as gender and age.

Genetic information	Grade	Gender	Age (years)
PAX6 missense mutation (c.1226-2A > G)	4	Male	43
PAX6 mutation (c.1191T(q227X))	3	Male	30
PAX6 missense mutation (c.607C > T)	4	Male	12
PAX6 missense mutation (c.1268A > T)	4	Female	50
PAX6 deletion (c.753_754delGC)	4	Female	46
PAX6 missense mutation (c.266A > C)	4	Female	36
PAX6 deletion (21q22.12q22. 236,472,360–39,889,694)x1	4	Female	2
PAX6 deletion (c.959_960delCA)	3	Female	41
Total	3 (25%); 4 (75%)	5 (62.5%) Female	32.50 ± 17.07 (2-50)

**Table 2:** Detailed information on the healthy corneal donors used in this study.

Donor number	Gender	Age (years)
1	Male	89
2	Female	64
3	Male	76
4	Female	82
5	Female	66
6	Female	58
7	Male	72
8	Female	72
9	Male	89
10	Female	64
11	Male	76
12	Female	82
Total	5 (41.67%) male	74.17 ± 10.06 (58–89)

### Primary stromal cell isolation

Biopsies were digested for 24 hours at 37 °C in KGM-3 medium (PromoCell GmbH, Germany) supplemented with collagenase A (1 mg/mL; Roche Diagnostics, #10103578001). The partially dissociated tissue was then gently triturated, following protocols described for human corneal stroma isolation, and passed through 40 µm Flowmi™ filters (Bel-Art, #H13680-0040) to obtain single-cell suspensions [26].

### Cell culture conditions

Filter-retained aggregates were treated with 0.05% trypsin-EDTA (Sigma-Aldrich® GmbH, Germany) for 5 minutes, neutralized with 5% fetal calf serum (FCS) in DMEM, pelleted (1,500 × g for 5 minutes), and seeded into 6-well plates containing complete DMEM supplemented with 5% FCS. Cultures were maintained at 37 °C in a humidified atmosphere of 5% CO₂ and 95% humidity. The medium was refreshed every 48–72 hours until approximately 70% confluence was reached.

### High-glucose exposure

The 70 mM for 48 h condition was selected based on our previous work as a supraphysiological challenge that induces robust early pathway remodeling without compromising cell viability, thereby enabling mechanistic interrogation [8]. Based on this, cells were incubated for 48 hours in DMEM containing either the standard 17.5 mM glucose concentration or a high-glucose formulation (70 mM), prepared by supplementing with D-glucose (Carl ROTH, Cat. X997).

### Apoptosis assay

Apoptosis was quantified using Annexin V-APC/propidium iodide (PI) dual staining (Invitrogen, Germany, #2734867). Following trypsinization, 1 × 10⁵ cells were resuspended in binding buffer and incubated with 5 µL Annexin V-APC for 15 minutes at room temperature in the dark. Cells were then counterstained with 5 µL PI immediately prior to analysis. Fluorescence was measured on a CytoFLEX flow cytometer (Beckman Coulter, CA, USA) using FL1 (585 nm) and FL4 (660 nm) detectors. Triplicate biological replicates were evaluated and classified as viable (Annexin V ⁻ /PI⁻), early apoptotic (Annexin V ⁺ /PI⁻), or late apoptotic/necrotic (Annexin V ⁺ /PI⁺).

### RNA extraction and cDNA synthesis

Total RNA was extracted using the NORGEN RNA Purification Plus Micro Kit, and RNA yield was measured spectrophotometrically (Analytik Jena AG, Jena, Germany). Samples were stored at −80 °C until further processing. cDNA was synthesized from 500 ng of total RNA using the OneTaq RT-PCR Kit (New England Biolabs Inc., Ipswich, USA) and stored at −20 °C until use.

### Quantitative real-time PCR

Primer sequences (Qiagen GmbH, Hilden, Germany; see Table 3) were used in combination with SYBR Green Master Mix (Vazyme, Nanjing, China). Each 10 µL reaction contained 5 µL master mix, 1 µL primer pair, 1 µL cDNA, and 3 µL nuclease-free water, and was run in duplicate on a QuantStudio 5 system (ThermoFisher Scientific™ GmbH, Germany). The cycling protocol was as follows: 95 °C for 10 s, 60 °C for 30 s, and 95 °C for 15 s, repeated for 40 cycles. Gene expression levels were normalized to β-glucuronidase (GUSB) using the 2^-ΔΔCt method, and results are presented as geometric mean ± SD.

**Table 3:** Specifications of Real-Time PCR Primers.

Target Transcript	Catalog Reference	Fragment Size (bp)	Transcript ID (NCBI)
BAX	QT00031192	111	NM_004324
BCL2	QT00025011	80	NM_000633
BID	QT00077833	98	NM_001196
CASP3	QT00023947	147	NM_004346
CASP7	QT00003549	149	NM_001227
CASP8	QT00052416	61	NM_001080124
CASP9	QT00036267	102	NM_001229
CASP10	QT00002394	91	NM_001206542
CDKN1A	QT00062090	79	NM_000389
CDKN1B	QT00998445	146	NM_004064
GUSB	QT00046046	96	NM_000181
BIRC5	QT00081186	101	NM_001168
TNF	QT00029162	98	NM_000594
XIAP	QT00042854	87	NM_001167

### Flow cytometry

LSCs and AN-LSCs were characterized in three independent experiments. Cells were detached using trypsin without EDTA (Merck, USA, No. T4424) and labeled with the primary antibodies listed in Table 4. Fluorochrome-conjugated secondary antibodies (Antibody Online, No. ABIN101988; Bio-Techne, No. F0118) were used as controls. Data acquisition was performed on a CytoFLEX flow cytometer, and analyses were conducted using CytExpert 2.6 software.

**Table 4:** Flow cytometry antibodies and detection reagents.

Marker Target	Clone/Type	Reactivity	Working Dilution	Vendor
BAX	E4U1V mAb (rabbit)	Human	1:100	Cell Signaling
BCL-2	Polyclonal (rabbit)	Human	1:100	Proteintech
BID	Polyclonal (rabbit)	Human	1:100	Proteintech
Caspase-3	Polyclonal (rabbit)	Human	1:100	Proteintech
Caspase-9	Polyclonal (rabbit)	Human	1:100	Cell Signaling
Caspase-10	Polyclonal (rabbit)	Human	1:100	Proteintech
Cleaved Caspase-7	D6H1 mAb (rabbit)	Human	1:100	Cell Signaling
Cleaved Caspase-8	E6H8S mAb (rabbit)	Human	1:100	Cell Signaling
Goat anti-Rabbit IgG	Preadsorbed	Secondary	1:500	Antibody Online
IgG1 isotype (mouse)	REAfinity	Secondary	1:50	Miltenyi Biotec
p21 (CDKN1A)	12D1 mAb (rabbit)	Human	1:100	Cell Signaling
p27 (CDKN1B)	SX53G8.5 mAb (mouse)	Human	1:100	Cell Signaling
Survivin	71G4B7 mAb (rabbit)	Human	1:100	Cell Signaling
TNF-α	D1G2 mAb (rabbit)	Human	1:100	Cell Signaling
XIAP	Polyclonal (rabbit)	Human	1:100	Proteintech

### Enzyme-linked immunosorbent assay

TNF-α concentrations in culture supernatants were measured in duplicate using the DuoSet® ELISA kit (R&D Systems, Bio-Techne, Minneapolis, USA, #DY210), following the manufacturer’s instructions. Absorbance at 450 nm was recorded on an Infinite F50 microplate reader (Tecan Group AG, Männedorf, Switzerland), and values were normalized to total protein content.

### Statistical analysis

Normality was assessed using the Shapiro–Wilk test. Concentration-dependent effects were evaluated by two-way ANOVA followed by Tukey’s post hoc multiple comparisons test (GraphPad Prism 9.0). qPCR data are presented as geometric mean ± SD, while flow cytometry and ELISA results are expressed as arithmetic mean ± SD. Statistical significance was defined as p < 0.05.

## Results

### Apoptotic ratio of cells

**Fig 1:**
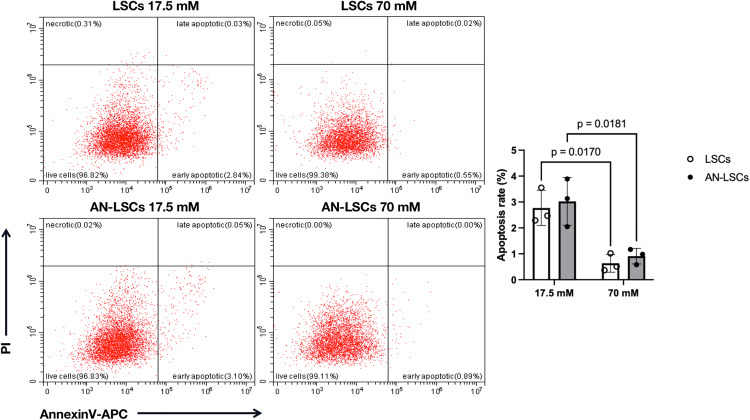
Apoptotic rates of limbal stromal cells (LSCs) and aniridia-LSCs (AN-LSCs), after treatment with 17.5 mM and 70 mM glucose for 48 hours. LSCs and AN-LSCs were cultured in medium containing either 17.5 mM or 70 mM glucose for 48 hours, then stained with Annexin V-APC/PI and analyzed by flow cytometry. Data represent the mean ± SD from three independent experiments. Statistical analysis was performed using two-way ANOVA followed by Tukey’s post hoc test; significant p values are indicated. Flow cytometry revealed that apoptosis was significantly reduced in both LSCs (p = 0.0170) and AN-LSCs (p = 0.0181) after 48 hours of treatment with 70 mM glucose compared to 17.5 mM.

Flow cytometry revealed that apoptosis was significantly reduced in both LSCs (p = 0.0170) and AN-LSCs (p = 0.0181) following 48 hours of treatment with 70 mM glucose compared to 17.5 mM (Fig 1).

### mRNA levels of apoptosis-related markers

**Fig 2:**
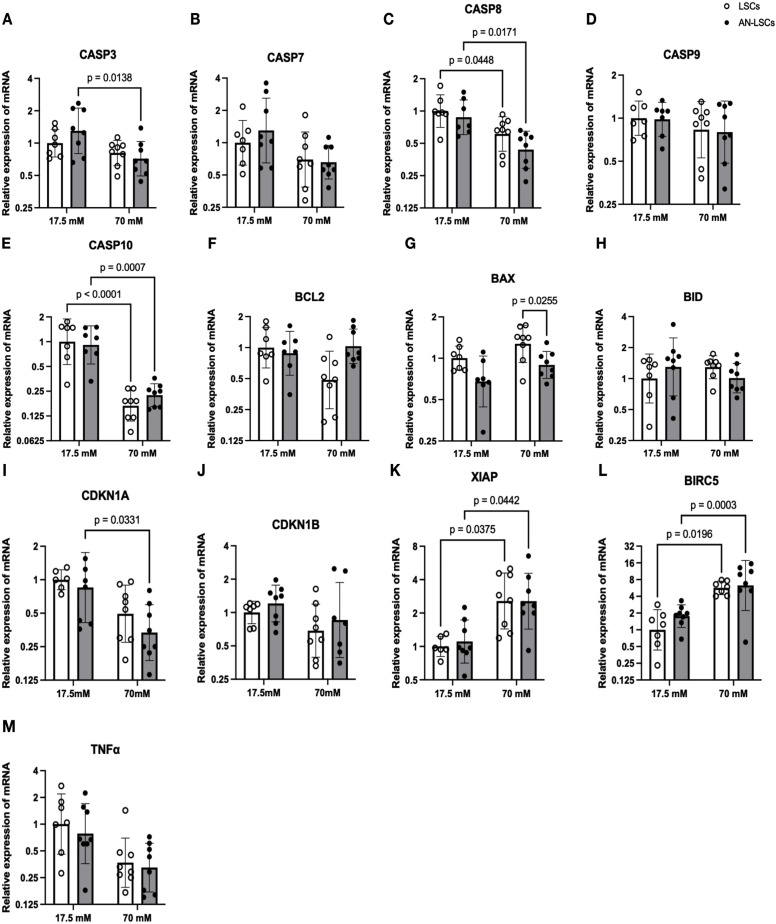
mRNA levels of CASP family markers (A-E), BCL2 family markers (F-H) and other apoptosis-related markers (I-M) in limbal stromal cells (LSCs) and aniridia-LSCs (AN-LSCs), after treatment with 17.5 mM and 70 mM glucose for 48 hours. *CASP3,* CASP*7,* CASP*8,* CASP*9* and *CASP10* mRNA levels (A-E), *BCL2, BAX* and *BID* mRNA levels (F-H) and *CDKN1A, CDKN1B, TNFα, XIAP* and *BIRC5* mRNA levels (I-M) are shown. Values are expressed on a logarithmic (log₂) scale as geometric mean ± geometric standard deviation. Statistical analysis was performed using two-way ANOVA followed by Tukey’s post hoc test; significant p values are indicated. Following 70 mM glucose treatment, *CASP8* and *CASP10* mRNA levels were significantly reduced in both LSCs (p = 0.0448) and AN-LSCs (p = 0.0171), while *XIAP* and *BIRC5* were significantly upregulated (p = 0.0375; p = 0.0442). In AN-LSCs, *CASP3* and *CDKN1A* mRNA levels were also significantly decreased under high glucose compared to 17.5 mM (p = 0.0331). Additionally, under 70 mM glucose conditions, *BAX* mRNA levels were significantly lower in AN-LSCs than in LSCs (p = 0.0255).

Following treatment with 70 mM glucose, *CASP8* and *CASP10* mRNA levels were significantly reduced in both LSCs (p ≤ 0.0448) and AN-LSCs (p ≤ 0.0171), while *XIAP* and *BIRC5* mRNA levels were significantly upregulated in both LSCs (p ≤ 0.0375) and AN-LSCs (p ≤ 0.0442). In AN-LSCs, the mRNA levels of *CASP3* and *CDKN1A* were also significantly decreased under 70 mM glucose compared to 17.5 mM (p ≤ 0.0331). Additionally, under 70 mM glucose conditions, *BAX* mRNA levels were significantly lower in AN-LSCs compared to LSCs (p = 0.0255) (Fig 2).

### Protein levels of apoptosis-related markers

**Fig 3:**
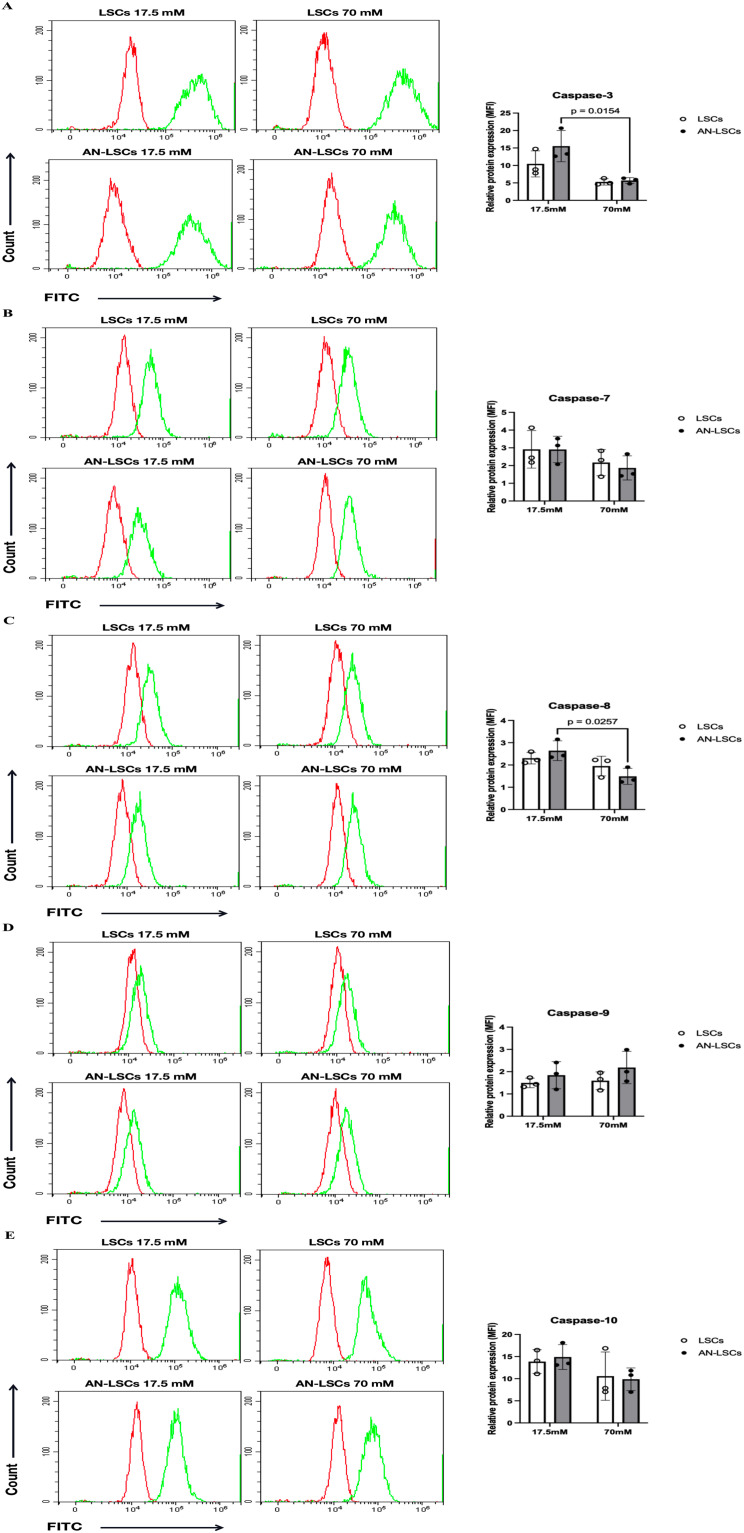
Protein levels of Caspase family markers in limbal stromal cells (LSCs) and aniridia-LSCs (AN-LSCs), after treatment with 17.5 mM and 70 mM glucose for 48 hours, using flow cytometry (A-E). Protein levels of Caspase-3, Caspase-7, Caspase-8, Caspase-9, and Caspase-10 are shown. Data represent the mean ± SD from three independent experiments. Statistical analysis was performed using two-way ANOVA followed by Tukey’s post hoc test; significant p values are indicated. Representative histograms display primary antibody staining in green and staining with the corresponding secondary antibody alone (negative control) in red. MFI: mean fluorescence intensity, normalized to the secondary antibody control. In AN-LSCs, the protein levels of Caspase-3 and Caspase-8 were significantly reduced following 70 mM glucose treatment compared to 17.5 mM (p ≤ 0.0257).

**Fig 4:**
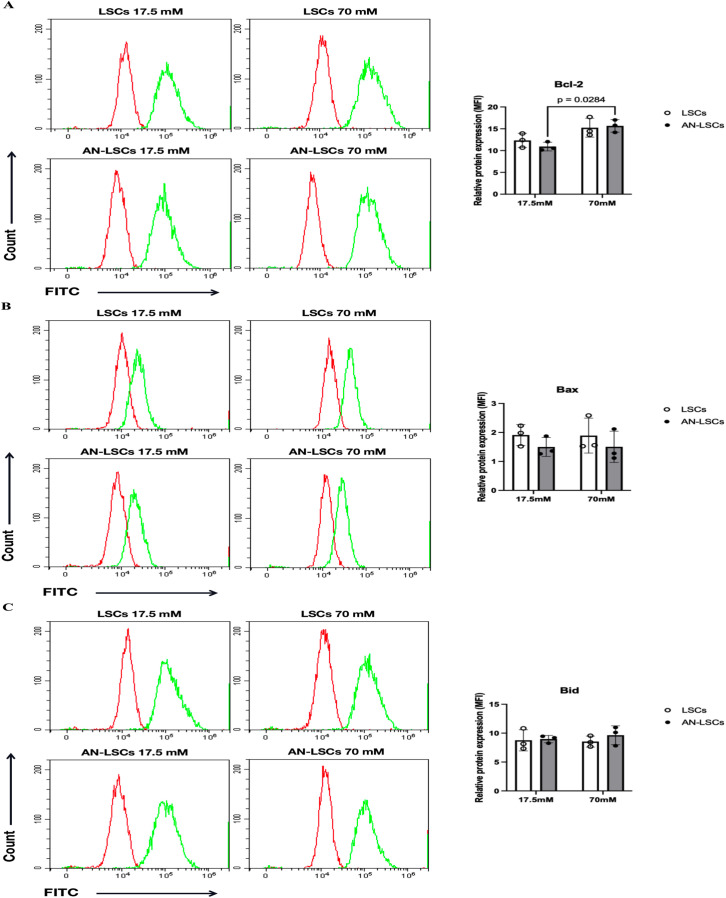
Protein levels of Bcl-2 family markers in limbal stromal cells (LSCs) and aniridia-LSCs (AN-LSCs), after treatment with 17.5 mM and 70 mM glucose for 48 hours, using flow cytometry (A-C). Protein levels of Bcl-2, Bax, and Bid are shown. Data represent the mean ± SD from three independent experiments. Statistical analysis was performed using two-way ANOVA followed by Tukey’s post hoc test; significant p values are indicated. Representative histograms display primary antibody staining in green, while staining with the corresponding secondary antibody alone (negative control) is shown in red. MFI: mean fluorescence intensity, normalized to the secondary antibody control. In AN-LSCs, Bcl-2 protein levels were significantly upregulated under 70 mM glucose treatment compared to 17.5 mM (p = 0.0284).

**Fig 5:**
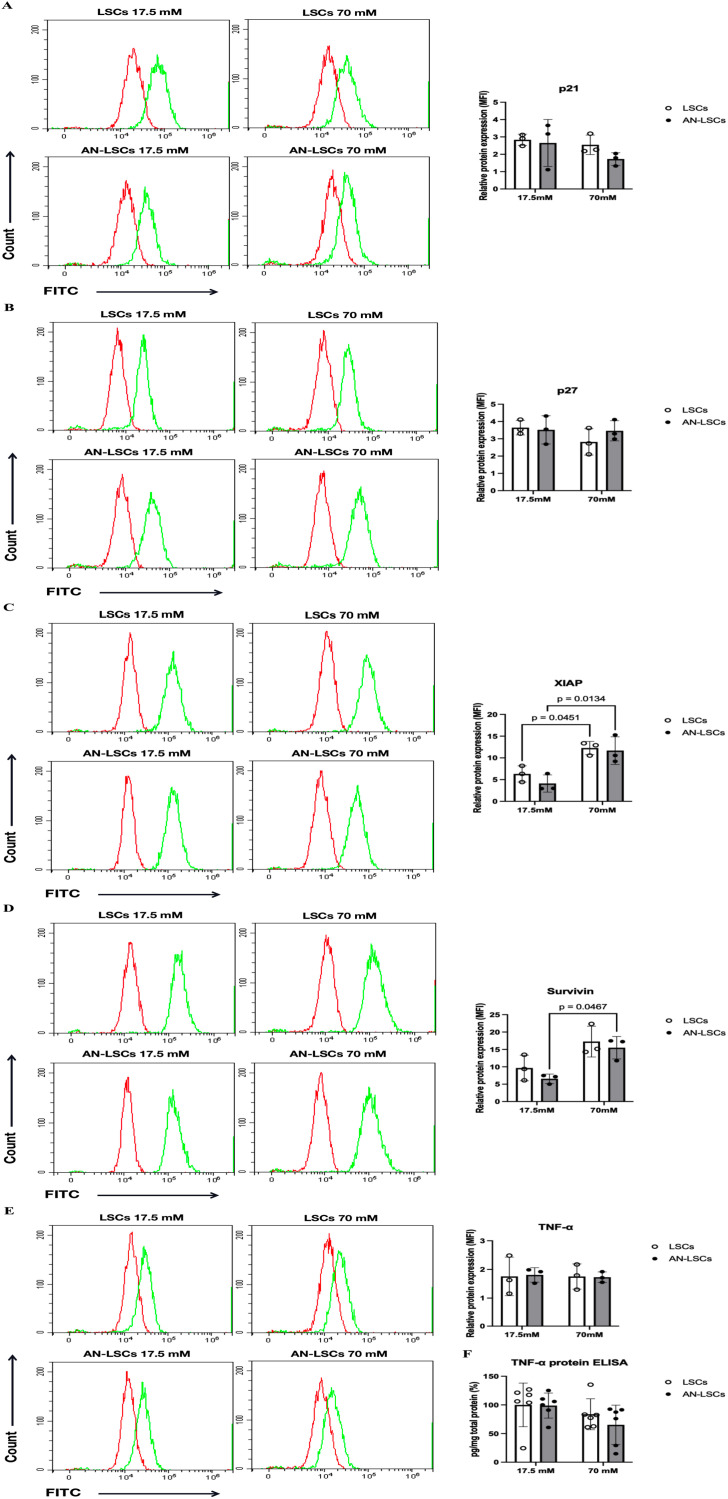
Protein levels of other apoptosis-related markers in limbal stromal cells (LSCs) and aniridia-LSCs (AN-LSCs), after treatment with 17.5 mM and 70 mM glucose for 48 hours, using flow cytometry (A-E) and ELISA (F). Protein levels of p21, p27, XIAP, TNF-α, and Survivin are shown. Data represent the mean ± SD from three independent experiments. Statistical analysis was performed using two-way ANOVA followed by Tukey’s post hoc test; significant p values are indicated. Representative histograms display primary antibody staining in green, with the corresponding secondary antibody alone (negative control) shown in red. MFI: mean fluorescence intensity, normalized to the secondary antibody control. Following 70 mM glucose treatment, XIAP protein levels increased significantly in both LSCs (p = 0.0451) and AN-LSCs (p = 0.0134), while Survivin protein levels showed a significant rise in AN-LSCs (p = 0.0467). Nevertheless, none of the other analyzed protein levels, assessed by flow cytometry or ELISA, changed significantly (p ≥ 0.2239).

In AN-LSCs, the protein levels of Caspase-3 and Caspase-8 were significantly reduced under 70 mM glucose treatment compared to 17.5 mM (p = 0.0154 and p = 0.0257, respectively), while Bcl-2 was significantly upregulated (p = 0.0284). Following 70 mM glucose exposure, XIAP protein levels were significantly increased in both LSCs (p = 0.0451) and AN-LSCs (p = 0.0134), whereas Survivin levels showed a significant rise specifically in AN-LSCs (p = 0.0467). Nevertheless, none of the other analyzed protein levels, assessed by flow cytometry or ELISA, changed significantly (p ≥ 0.2239) (Figs 3, 4 and 5).

## Discussion

**Fig 6:**
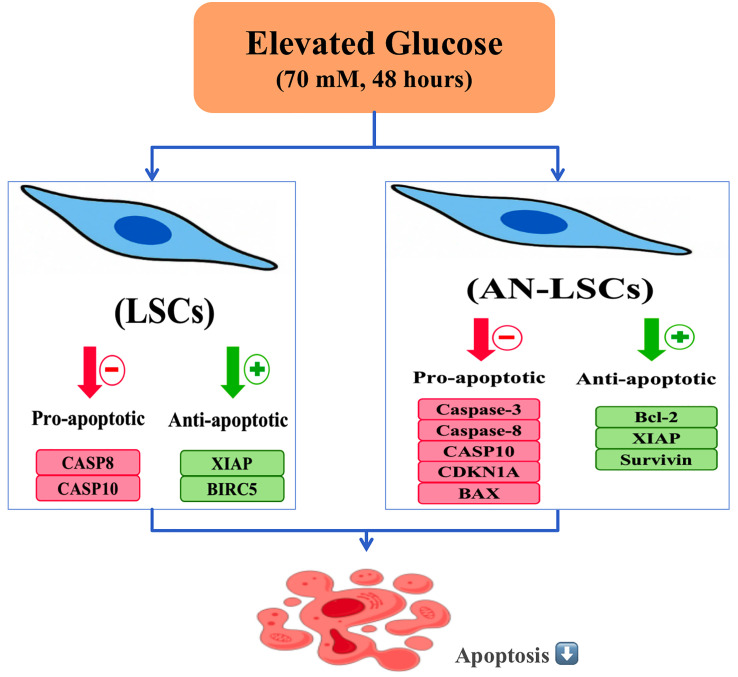
Acute high-glucose exposure induces anti-apoptotic signaling in human limbal stromal cells of healthy subjects and patients with congenital aniridia. Schematic representation of the effects of high glucose (70 mM, 48 hours) on apoptotic signaling in human limbal stromal cells (LSCs) and aniridia-LSCs (AN-LSCs). Elevated glucose suppresses the expression of pro-apoptotic factors, including CASP8, CASP10, BAX, and CASP3, while simultaneously enhancing the expression of anti-apoptotic molecules such as BCL-2, XIAP, and Survivin. In AN-LSCs, these effects are even more pronounced, with notable reductions in executioner Caspase-3 and the cell cycle inhibitor p21 (CDKN1A), indicating a more robust anti-apoptotic adaptation in these cells. This coordinated molecular shift contributes to the inhibition of apoptosis and promotes cell survival, particularly in congenital aniridia-derived limbal stromal cells. The figure illustrates the shift in balance from apoptotic signaling toward a survival-promoting profile under acute metabolic stress.

Our study demonstrates that exposure to high glucose (70 mM for 48 hours) induces a significant anti-apoptotic shift in primary human limbal stromal cells (LSCs) and congenital aniridia-derived limbal stromal cells (AN-LSCs). Compared to physiological glucose conditions (17.5 mM), elevated glucose markedly reduced apoptosis, as confirmed by Annexin V/PI analysis, and was associated with distinct changes in apoptotic regulators that favor cell survival. Notably, these anti-apoptotic responses were more pronounced in AN-LSCs. Specifically, high glucose exposure led to reduced expression of initiator caspases involved in the extrinsic apoptotic pathway (Caspase-8 and Caspase-10) and decreased levels of the intrinsic mitochondrial pro-apoptotic factor BAX, while simultaneously upregulating the anti-apoptotic protein BCL-2. Additionally, the anti-apoptotic proteins XIAP and Survivin were significantly increased at both the transcriptional and protein levels. This coordinated downregulation of pro-apoptotic pathways likely accounts for the observed decrease in apoptosis. In AN-LSCs, these effects were even more pronounced, with notable reductions in executioner Caspase-3 and the cell cycle inhibitor p21 (CDKN1A), indicating a more robust anti-apoptotic adaptation in these cells (Fig 6).

While our data demonstrate a direct reduction in apoptosis and a coordinated modulation of core apoptotic regulators, we acknowledge that the overall decrease in cell death could, in theory, also be influenced by other cellular states, such as senescence or cell cycle arrest. The observed downregulation of the cell cycle inhibitor p21 (CDKN1A) in AN-LSCs suggests that cell cycle arrest is unlikely to be the primary driver of our findings. However, a comprehensive assessment of senescence markers or detailed cell cycle profiling was beyond the scope of the present study. Thus, while we describe a clear “anti-apoptotic shift” based on the measured parameters, future work will be important to characterize the broader phenotypic state of these cells under metabolic stress.

The more pronounced reduction in apoptosis and stronger upregulation of anti-apoptotic proteins observed in AN-LSCs may reflect a more robust adaptive survival response to acute metabolic stress. An alternative interpretation is that AN-LSCs exhibit greater intrinsic sensitivity to the stressor, thereby requiring a stronger compensatory anti-apoptotic reaction to maintain viability. Our previous findings of altered baseline signaling in AN-LSCs [8,12] support the concept of a fundamentally different stress threshold in these cells. Discriminating between these possibilities will require future studies assessing early markers of cellular stress and damage immediately after high-glucose exposure.

Based on our current findings and our previous study, we propose an early adaptive–survival hypothesis to explain the reduced apoptosis observed under the 70 mM/48 h high-glucose condition. Specifically, we suggest that extrinsic apoptotic signaling is attenuated, as high glucose reduces NF-κB and TGF-β1 activity and is accompanied by CASP8/10 downregulation, including lower Caspase-8 protein levels in AN-LSCs, thereby dampening death-receptor–mediated apoptosis. In parallel, mitochondrial apoptotic pathways appear to be restrained through an antioxidant shift characterized by Nrf2 and CAT upregulation and an increased BCL-2/BAX ratio, particularly in AN-LSCs. Furthermore, executioner activity is limited by elevated XIAP and Survivin expression together with reduced Caspase-3 levels in AN-LSCs. Collectively, these coordinated alterations suggest that high glucose triggers a transient anti-apoptotic state at 48 h, representing an early adaptive response that, according to our prior work [8], attenuates or reverses by 72 h.

These findings initially appear inconsistent with prior reports that predominantly link hyperglycemia to increased apoptosis in ocular cells like retinal pericytes and corneal endothelial cells [25,27–29]. However, our observations might instead reflect a protective, short-term adaptive response of limbal stromal cells to acute metabolic stress. This interpretation is supported by our previous work, which demonstrated that acute high-glucose exposure significantly downregulated pro-inflammatory and pro-fibrotic signaling pathways (TGF-β1 and NF-κB) while simultaneously upregulating antioxidant defense mechanisms (Nrf2 and catalase), thereby creating a microenvironment that mitigates oxidative stress and inflammatory damage [8]. The suppressed extrinsic apoptotic signaling observed in our study—evidenced by reduced Caspase-8 and Caspase-10 expression—is consistent with diminished inflammatory cytokine activity [8], likely a consequence of decreased NF-κB signaling. Building on our previous work demonstrating glucose-induced remodeling of NF-κB—a critical transcription factor controlling TNFα expression—examining TNFα itself was both a logical and essential next step. This analysis enabled us to assess the functional consequences of these signaling alterations within a key death receptor pathway. Notably, although TNFα levels remained unchanged, its downstream effectors (CASP8/10) were suppressed. This finding reinforces the idea that cells are not simply shutting down the pathway, but are instead mounting a refined, adaptive response that selectively uncouples survival mechanisms from inflammatory signaling. Including TNFα was therefore crucial to fully elucidate the nature of this anti-apoptotic shift. Similarly, the intrinsic mitochondrial apoptotic pathway appears stabilized by a shift in the BCL-2/BAX ratio toward anti-apoptotic dominance, which would inhibit cytochrome c release [30]. Elevated levels of XIAP and Survivin further contribute to this anti-apoptotic state by blocking caspase activation, thereby enhancing cellular resistance to apoptosis [31].

Interestingly, reduced NF-κB activity did not negatively impact cell survival; rather, decreased NF-κB likely suppressed pro-apoptotic inflammatory signaling, while compensatory mechanisms—possibly involving Nrf2-dependent antioxidant responses [8]—elevated survival factors such as XIAP and Survivin, ultimately promoting cell survival [32]. This intricate remodeling of signaling pathways suggests that high glucose induces a coordinated cellular survival response, consistent with our earlier studies on TGF-β1 and NF-κB dynamics, which similarly demonstrated initial anti-inflammatory and pro-survival adjustments [8]. In contrast, chronic hyperglycemia is well-documented to cause cumulative cellular damage, oxidative stress, inflammation, and apoptosis, as seen in diabetic ocular complications [33–35]. Notably, however, acute high-glucose exposure has been shown in certain cell types, such as cardiac fibroblasts, to stimulate proliferation and upregulate anti-apoptotic gene expression, aligning with our observations [36]. This suggests that fibroblastic cells from various tissues may deploy transient protective strategies against metabolic stress, although these responses may not persist under chronic conditions. Indeed, our previous results indicated a shift toward pro-inflammatory signaling and diminished antioxidant defenses when glucose exposure was extended beyond 72 hours, highlighting a potential eventual loss of adaptive capacity under prolonged stress [8].

These findings complement our previous studies examining LSCs and AN-LSCs apoptosis under hypoxic and inflammatory stress. Under CoCl₂-induced hypoxia, AN-LSCs showed elevated apoptosis, with downregulation of anti-apoptotic markers (BCL2, XIAP, Survivin) and increased Caspase-3/9 activity [37]. Similarly, LPS-induced inflammation significantly upregulated pro-apoptotic genes (CASP3/7/10, TNF-α) and further suppressed BCL2 and XIAP in AN-LSCs [38]. In contrast, high-glucose exposure in the present study led to a reduction in apoptosis, accompanied by upregulation of XIAP and Survivin and downregulation of CASP8/10, suggesting a transient anti-apoptotic adaptation. While all three stressors revealed AN-LSC hypersensitivity, only metabolic stress elicited a compensatory survival response—highlighting how different environmental insults trigger distinct apoptotic programs. Together, these results underscore the vulnerability of AN-LSCs to cellular stress and suggest that stress-specific modulation of apoptosis may offer tailored therapeutic opportunities in AAK.

This study has several technical limitations that warrant consideration. First, most notably the age difference between congenital aniridia patients and cadaveric donors. Although we aimed to investigate intrinsic differences between limbal stromal cells from healthy and aniridic eyes under identical culture conditions, using low-passage cells to minimize senescence-related effects, the potential influence of age-associated factors cannot be entirely excluded. Second, while the use of a limited number of donor samples is acceptable for an initial exploratory investigation, it may constrain the generalizability of our findings. Expanding the sample cohort will be essential to capture inter-individual variability in gene expression patterns. Third, while Annexin V/PI staining provides a functional measure of apoptosis, we did not include cleaved PARP or caspase activity assays in this study due to the limited yield of primary cells and the broad molecular endpoint profiling performed. Future work will incorporate cleaved PARP and caspase-3/7, −8, and −9 activity assays (including z-VAD-fmk rescue experiments), along with mitochondrial integrity assessments and IAP perturbation studies, to functionally validate the proposed early adaptive–survival model. Fourth, in our previous study, the measured osmolarities were 309 mOsm/kg for 17.5 mM glucose and 362 mOsm/kg for 70 mM glucose in DMEM [8]. These values fall within the range commonly used in cell culture models of hyperglycemia, where glucose concentrations typically range from 17.5 to 25 mM, above the physiological norm of 5–7 mM [39–41]. Our experimental conditions therefore represent moderate osmotic elevations rather than extreme hyperosmotic stress. For comparison, Petronini et al. reported that protein synthesis in chick embryo fibroblasts begins to decline at approximately 400 mOsm/kg, while Feyerabend found that cell viability is maintained below this threshold but necrosis increases above it [42,43]. Accordingly, the osmolarity range of 309–362 mOsm/kg used in our study remains below levels known to induce nonspecific osmotic toxicity, supporting the interpretation that the observed cellular responses primarily reflect biological effects of glucose metabolism rather than osmotic artifacts. Nevertheless, while the observed anti-apoptotic shifts are consistent with specific metabolic stress responses, the potential contribution of hyperosmolarity cannot be completely ruled out within the current experimental framework. Future studies will therefore include an osmotic control (e.g., mannitol supplementation) to more precisely distinguish metabolic from osmotic effects. Fifth, a key limitation of this study is the focus on a single, 48-hour time point. Our previous work demonstrated that the initial suppression of TGF-β1 and NF-κB at 48 hours of high-glucose exposure can rebound or even reverse with prolonged exposure (72 hours) [8]. It is therefore plausible that the protective anti-apoptotic shift observed here is transient. A critical avenue for future research will be to perform a time-course analysis of apoptotic markers to determine if and when this survival response is superseded by a pro-apoptotic phenotype, thereby linking prolonged metabolic stress more directly to the progression of limbal dysfunction in AAK. Sixth, the absence of functional validation of key molecules—such as through targeted manipulation of CASP8 or BIRC5 using CRISPR-based approaches—limits our ability to draw causal inferences from the observed expression changes. Seventh, the acute high-glucose exposure model employed here (48 hours), although effective for capturing early stress responses, does not fully replicate the sustained metabolic imbalance and chronic inflammatory milieu (e.g., persistent TNF-α elevation) that characterize AAK. This design was chosen specifically to model an acute stressor to reveal early signaling directionality; future studies will include broader dose–response and time-course experiments to emulate chronic exposures. Such multifactorial interactions are likely to play a critical role in driving the long-term deterioration of the limbal microenvironment. Finally, although TNF-α was analyzed as a representative cytokine of extrinsic apoptosis, other mediators such as IFN-γ and IL-1β—acting through STAT1 and MyD88/NF-κB pathways—may influence apoptotic tone and exhibit distinct kinetic profiles. Upcoming studies will implement multiplex cytokine profiling over a 6–72hour window, assess downstream pathway activation (e.g., phosphorylated STAT1), and combine cytokine add-back or neutralization experiments with death-receptor phenotyping to clarify their role in the anti-apoptotic shift reported here.

Future studies should aim to validate these adaptive responses in vivo, investigate the effects of prolonged metabolic stress, and explore interactions with additional stressors such as inflammation and hypoxia. Ultimately, harnessing the early adaptive protective mechanisms identified in this study could pave the way for novel therapeutic strategies to manage AAK.

## Conclusions

This study demonstrates that acute high-glucose exposure (70 mM for 48 hours) significantly suppresses apoptosis in human limbal stromal cells, with a more pronounced effect observed in cells derived from aniridia patients. This response is characterized by the downregulation of key pro-apoptotic factors and the upregulation of anti-apoptotic molecules. These findings indicate that limbal stromal cells may activate intrinsic survival pathways as an early adaptive response to metabolic stress, providing new insights into the pathogenesis of AAK and laying a potential foundation for the development of targeted therapeutic strategies.

## Supporting information

S1 File Raw data apoptosis, mRNA and protein. (PDF)
