# Supplementary material for: Elevated anti-apoptotic shift in primary human aniridia limbal stromal cells following 48 hours supraphysiological glucose exposure, in vitro
Source: PLoS One. 2026 Jan 12;21(1):e0340117. doi: 10.1371/journal.pone.0340117 (PMC12795463; doi:10.1371/journal.pone.0340117)
Supplement: S1 File — (PDF) [file pone.0340117.s001.pdf]

CDKN1A(p21)

Sample Name (ctr/48h-17.5mM)	Ct Mean CDKN1A	ct Mean TBP crt	ct Mean GUSB crt	Mean GUSB/TBP	ΔCt (Ct-Mean-GUSB/TE	ΔΔCt (Mean Ctrl - DCt)	Fold Change
LFC 153/P7	19.124	22.912	22.266	22.59	-3.47	0.04	1.02
LFC 396/P3	19.943	23.735	22.555	23.14	-3.20	-0.23	0.85
LFC 416/P4	19.650	23.874	23.031	23.45	-3.80	0.37	1.29
LFC 427/P4	20.685	24.024	23.335	23.68	-3.00	-0.43	0.74
LFC 420/P4	19.565	23.703	22.296	23.00	-3.43	0.00	1.00
LFC 422/P4	19.728	23.732	23.083	23.41	-3.68	0.25	1.19
					-3.430	0.000	1.000
**Mittelwert crt**	**19.78**	**23.66**	**22.76**	**23.21**	**-3.430**	0.000	**1.000**

Sample Name (ctr/48h-70mM)	Ct Mean CDKN1A	ct Mean TBP crt	ct Mean GUSB crt	Mean GUSB/TBP	ΔCt (Ct-Mean-GUSB/TE	ΔΔCt (Mean Ctrl - DCt)	Fold Change
LFC 491	23.635	25.515	23.809	24.66	-1.03	-2.40	0.19
LFC 492	22.800	26.584	24.521	25.55	-2.75	-0.68	0.63
LFC 777	23.851	26.439	24.614	25.53	-1.68	-1.75	0.30
LFC 471	22.204	24.845	22.892	23.87	-1.67	-1.76	0.29
LFC 467	20.705	24.522	23.640	24.08	-3.38	-0.05	0.96
LFC 392	21.861	25.440	23.355	24.40	-2.54	-0.89	0.54
LFC 486	23.125	27.170	25.655	26.41	-3.29	-0.14	0.91
LFC 481	22.028	25.954	23.974	24.96	-2.94	-0.49	0.71
					-2.407		0.492
**Mittelwert crt**	**22.53**	**25.81**	**24.06**	**24.93**	**-2.407**	-1.023	**0.492**

Sample Name (aniridia/48h-17.5mM)	Ct Mean CDKN1A	ct Mean TBP crt	ct Mean GUSB crt	Mean GUSB/TBP	ΔCt (Ct-Mean-GUSB/TE	ΔΔCt (Mean Ctrl - DCt)	Fold Change
AN-LFC12/P5	19.042	23.519	22.681	23.10	-4.06	0.63	1.55
AN-LFC13/P5	19.119	22.550	22.107	22.33	-3.21	-0.22	0.86
AN-LFC 5/P4	20.688	25.567	22.614	24.09	-3.40	-0.03	0.98
AN-LFC6/P3	19.089	23.28	22.385	22.83	-3.74	0.31	1.24
AN-LFC 7/P3	20.088	22.556	22.025	22.29	-2.20	-1.23	0.43
AN-LFC 8/P4	21.814	23.940	23.884	23.91	-2.10	-1.33	0.40
AN-LFC10/P4	19.216	23.739	24.482	24.11	-4.89	1.46	2.76
AN-LFC12/P5	21.280	23.508	22.997	23.25	-1.97	-1.46	0.36
					-3.197		0.851
**Mittelwert crt**	**20.04**	**23.58**	**22.90**	**23.24**	**-3.197**	-0.232	**0.851**
**SD**							

Sample Name (aniridia/48h-70mM)	Ct Mean CDKN1A	ct Mean TBP crt	ct Mean GUSB crt	Mean GUSB/TBP	ΔCt (Ct-Mean-GUSB/TE	ΔΔCt (Mean Ctrl - DCt)	Fold Change
AN-LFC15	19.357	22.286	21.867	22.08	-2.72	-0.71	0.61
AN-LFC 5	21.354	24.533	22.916	23.72	-2.37	-1.06	0.48
AN-LFC14	24.117	26.203	24.863	25.53	-1.42	-2.01	0.25
AN-LFC16	24.066	25.501	23.790	24.65	-0.58	-2.85	0.14
AN-LFC17	23.104	25.140	23.551	24.35	-1.24	-2.19	0.22
AN-LFC 6	24.238	27.054	25.264	26.16	-1.92	-1.51	0.35
AN-LFC 1	22.902	25.548	23.151	24.35	-1.45	-1.98	0.25
AN-LFC 8	24.559	28.620	26.720	27.67	-3.11	-0.32	0.80
					-1.851		0.335
**Mittelwert Aniridie**	**22.96**	**25.61**	**24.02**	**24.81**	**-1.851**	-1.579	**0.335**
**SD**							

TNFα

Sample Name (ctr/48h-17.5mM)	Ct Mean TNFα	ct Mean TBP crt	ct Mean GUSB crt	Mean GUSB/TBP	ΔCt (Ct-Mean-GUSB/TE	ΔΔCt (Mean Ctrl - DCt)	Fold Change
LFC 135/P7							
LFC 153/P7	29.810	22.912	22.266	22.59	7.22	-0.02	0.99
LFC 396/P3	32.201	23.735	22.555	23.14	9.06	-1.86	0.28
LFC 416/P4	30.043	23.874	23.031	23.45	6.59	0.61	1.53
LFC 427/P4	30.826	24.024	23.335	23.68	7.15	0.05	1.04
LFC 428/P4	33.339	26.927	27.022	26.97	6.36	0.83	1.78
LFC 420/P4	31.249	23.703	22.296	23.00	8.25	-1.05	0.48
LFC 422/P4	29.176	23.732	23.083	23.41	5.77	1.43	2.70
					7.200	0.000	1.000
**Mittelwert crt**	**30.95**	**24.13**	**23.37**	**23.75**	**7.200**	0.000	**1.000**

Sample Name (ctr/48h-70mM)	Ct Mean TNFα	ct Mean TBP crt	ct Mean GUSB crt	Mean GUSB/TBP	ΔCt (Ct-Mean-GUSB/TE	ΔΔCt (Mean Ctrl - DCt)	Fold Change
LFC 491	34.393	25.515	23.809	24.66	9.73	-2.53	0.17
LFC 492	34.343	26.584	24.521	25.55	8.79	-1.59	0.33
LFC 777	33.799	26.439	24.614	25.53	8.27	-1.07	0.48
LFC 471	32.951	24.845	22.892	23.87	9.08	-1.88	0.27
LFC 467	33.067	24.522	23.640	24.08	8.99	-1.79	0.29
LFC 392	32.744	25.440	23.355	24.40	8.35	-1.15	0.45
LFC 486	33.092	27.170	25.655	26.41	6.68	0.52	1.43
LFC 481	34.157	25.954	23.974	24.96	9.19	-1.99	0.25
					8.635		0.370
**Mittelwert**	**33.57**	**25.81**	**24.06**	**24.93**	**8.635**	-1.435	**0.370**

Sample Name (aniridia/48h-17.5mM)	Ct Mean TNFα	ct Mean TBP crt	ct Mean GUSB crt	Mean GUSB/TBP	ΔCt (Ct-Mean-GUSB/TE	ΔΔCt (Mean Ctrl - DCt)	Fold Change
AN-LFC12/P5	29.850	23.519	22.681	23.10	6.75	0.45	1.37
AN-LFC13/P5	31.991	22.550	22.107	22.33	9.66	-2.46	0.18
AN-LFC 5/P4	32.027	25.567	22.614	24.09	7.94	-0.74	0.60
AN-LFC6/P3	29.392	23.28	22.385	22.83	6.56	0.64	1.56
AN-LFC 7/P3	30.071	22.556	22.025	22.29	7.78	-0.58	0.67
AN-LFC 8/P4	29.946	23.940	23.884	23.91	6.03	1.17	2.24
AN-LFC10/P4	31.869	23.739	24.482	24.11	7.76	-0.56	0.68
AN-LFC12/P5	31.177	23.508	22.997	23.25	7.93	-0.73	0.60
					7.551		0.784
**Mittelwert crt**	**30.79**	**23.58**	**22.90**	**23.24**	**7.551**	-0.351	**0.784**
**SD**							

Sample Name (aniridia/48h-70mM)	Ct Mean TNFα	ct Mean TBP crt	ct Mean GUSB crt	Mean GUSB/TBP	ΔCt (Ct-Mean-GUSB/TE	ΔΔCt (Mean Ctrl - DCt)	Fold Change
AN-LFC15	31.934	22.286	21.867	22.08	9.86	-2.66	0.16
AN-LFC 5	33.702	24.533	22.916	23.72	9.98	-2.78	0.15
AN-LFC14	33.945	26.203	24.863	25.53	8.41	-1.21	0.43
AN-LFC16	34.296	25.501	23.790	24.65	9.65	-2.45	0.18
AN-LFC17	33.311	25.140	23.551	24.35	8.97	-1.77	0.29
AN-LFC 6	33.829	27.054	25.264	26.16	7.67	-0.47	0.72
AN-LFC 1	32.486	25.548	23.151	24.35	8.14	-0.94	0.52
AN-LFC 8	35.572	28.620	26.720	27.67	7.90	-0.70	0.61
					8.821		0.325
**Mittelwert Aniridie**	**33.63**	**25.61**	**24.02**	**24.81**	**8.821**	-1.622	**0.325**
**SD**							

Casp3

Sample Name (ctr/48h-17.5mM)	Ct Mean Casp3	ct Mean TBP crt	ct Mean GUSB crt	Mean GUSB/TBP	ΔCt (Ct-Mean-GUSB/TE	ΔΔCt (Mean Ctrl - DCt)	Fold Change
LFC 153/P7	23.089	22.912	22.266	22.59	0.50	-0.48	0.72
LFC 396/P3	23.573	23.735	22.555	23.14	0.43	-0.41	0.75
LFC 416/P4	23.508	23.874	23.031	23.45	0.06	-0.04	0.97
LFC 427/P4	23.255	24.024	23.335	23.68	-0.42	0.44	1.36
LFC 428/P4	26.378	26.927	27.022	26.97	-0.60	0.62	1.53
LFC 420/P4	23.315	23.703	22.296	23.00	0.32	-0.30	0.81
LFC 422/P4	23.261	23.732	23.083	23.41	-0.15	0.17	1.12
					0.019	0.000	1.000
**Mittelwert crt**	**23.77**	**24.13**	**23.37**	**23.75**	**0.019**	0.000	**1.000**

Sample Name (ctr/48h-70mM)	Ct Mean Casp3	ct Mean TBP crt	ct Mean GUSB crt	Mean GUSB/TBP	ΔCt (Ct-Mean-GUSB/TE	ΔΔCt (Mean Ctrl - DCt)	Fold Change
LFC 491	25.700	25.515	23.809	24.66	1.04	-1.02	0.49
LFC 492	25.709	26.584	24.521	25.55	0.16	-0.14	0.91
LFC 777	25.364	26.439	24.614	25.53	-0.16	0.18	1.13
LFC 471	24.148	24.845	22.892	23.87	0.28	-0.26	0.83
LFC 467	24.181	24.522	23.640	24.08	0.10	-0.08	0.95
LFC 392	24.756	25.440	23.355	24.40	0.36	-0.34	0.79
LFC 486	26.493	27.170	25.655	26.41	0.08	-0.06	0.96
LFC 481	25.700	25.954	23.974	24.96	0.74	-0.72	0.61
					0.323		0.810
**Mittelwert**	**25.26**	**25.81**	**24.06**	**24.93**	**0.323**	-0.304	**0.810**

Sample Name (aniridia/48h-17.5mM)	Ct Mean Casp3	ct Mean TBP crt	ct Mean GUSB crt	Mean GUSB/TBP	ΔCt (Ct-Mean-GUSB/TE	ΔΔCt (Mean Ctrl - DCt)	Fold Change
AN-LFC12/P5	22.796	23.519	22.681	23.10	-0.30	0.32	1.25
AN-LFC13/P5	22.940	22.550	22.107	22.33	0.61	-0.59	0.66
AN-LFC 5/P4	22.876	25.567	22.614	24.09	-1.21	1.23	2.35
AN-LFC6/P3	23.325	23.28	22.385	22.83	0.49	-0.47	0.72
AN-LFC 7/P3	21.858	22.556	22.025	22.29	-0.43	0.45	1.37
AN-LFC 8/P4	22.902	23.940	23.884	23.91	-1.01	1.03	2.04
AN-LFC10/P4	23.061	23.739	24.482	24.11	-1.05	1.07	2.10
AN-LFC12/P5	23.266	23.508	22.997	23.25	0.01	0.00	1.00
					-0.361		1.302
**Mittelwert crt**	**22.88**	**23.58**	**22.90**	**23.24**	**-0.361**	0.380	**1.302**
**SD**							

Sample Name (aniridia/48h-70mM)	Ct Mean Casp3	ct Mean TBP crt	ct Mean GUSB crt	Mean GUSB/TBP	ΔCt (Ct-Mean-GUSB/TE	ΔΔCt (Mean Ctrl - DCt)	Fold Change
AN-LFC15	23.275	22.286	21.867	22.08	1.20	-1.18	0.44
AN-LFC 5	24.564	24.533	22.916	23.72	0.84	-0.82	0.57
AN-LFC14	26.482	26.203	24.863	25.53	0.95	-0.93	0.52
AN-LFC16	24.876	25.501	23.790	24.65	0.23	-0.21	0.86
AN-LFC17	23.900	25.140	23.551	24.35	-0.45	0.46	1.38
AN-LFC 6	26.734	27.054	25.264	26.16	0.58	-0.56	0.68
AN-LFC 1	24.927	25.548	23.151	24.35	0.58	-0.56	0.68
AN-LFC 8	27.734	28.620	26.720	27.67	0.06	-0.05	0.97
					0.499		0.717
**Mittelwert Aniridie**	**25.31**	**25.61**	**24.02**	**24.81**	**0.499**	-0.480	**0.717**
**SD**							

Casp7

Sample Name (ctr/48h-17.5mM)	Ct Mean Casp7	ct Mean TBP crt	ct Mean GUSB crt	Mean GUSB/TBP	ΔCt (Ct-Mean-GUSB/TE	ΔΔCt (Mean Ctrl - DCt)	Fold Change
LFC 153/P7	28.218	22.912	22.266	22.59	5.63	1.12	2.17
LFC 396/P3	29.815	23.735	22.555	23.14	6.67	0.07	1.05
LFC 416/P4	30.84	23.874	23.031	23.45	7.39	-0.64	0.64
LFC 427/P4	30.057	24.024	23.335	23.68	6.38	0.37	1.29
LFC 428/P4	33.914	26.927	27.022	26.97	6.94	-0.19	0.87
LFC 420/P4	29.496	23.703	22.296	23.00	6.50	0.25	1.19
LFC 422/P4	31.123	23.732	23.083	23.41	7.72	-0.97	0.51
					6.745	0.000	1.000
**Mittelwert crt**	**30.49**	**24.13**	**23.37**	**23.75**	**6.745**	0.000	**1.000**

Sample Name (ctr/48h-70mM)	Ct Mean Casp7	ct Mean TBP crt	ct Mean GUSB crt	Mean GUSB/TBP	ΔCt (Ct-Mean-GUSB/TE	ΔΔCt (Mean Ctrl - DCt)	Fold Change
LFC 491	32.837	25.515	23.809	24.66	8.18	-1.43	0.37
LFC 492	32.414	26.584	24.521	25.55	6.86	-0.12	0.92
LFC 777	34.077	26.439	24.614	25.53	8.55	-1.81	0.29
LFC 471	31.358	24.845	22.892	23.87	7.49	-0.74	0.60
LFC 467	31.332	24.522	23.640	24.08	7.25	-0.51	0.70
LFC 392	30.286	25.440	23.355	24.40	5.89	0.86	1.81
LFC 486	32.941	27.170	25.655	26.41	6.53	0.22	1.16
LFC 481	32.359	25.954	23.974	24.96	7.39	-0.65	0.64
					7.267		0.696
**Mittelwert**	**32.20**	**25.81**	**24.06**	**24.93**	**7.267**	-0.522	**0.696**

Sample Name (aniridia/48h-17.5mM)	Ct Mean Casp7	ct Mean TBP crt	ct Mean GUSB crt	Mean GUSB/TBP	ΔCt (Ct-Mean-GUSB/TE	ΔΔCt (Mean Ctrl - DCt)	Fold Change
AN-LFC12/P5	29.655	23.519	22.681	23.10	6.56	0.19	1.14
AN-LFC13/P5	29.026	22.550	22.107	22.33	6.70	0.05	1.03
AN-LFC 5/P4	31.611	25.567	22.614	24.09	7.52	-0.78	0.58
AN-LFC6/P3	30.359	23.28	22.385	22.83	7.53	-0.78	0.58
AN-LFC 7/P3	28.056	22.556	22.025	22.29	5.77	0.98	1.97
AN-LFC 8/P4	29.065	23.940	23.884	23.91	5.15	1.59	3.01
AN-LFC10/P4	29.008	23.739	24.482	24.11	4.90	1.85	3.60
AN-LFC12/P5	30.054	23.508	22.997	23.25	6.80	-0.06	0.96
					6.365		1.302
**Mittelwert crt**	**29.60**	**23.58**	**22.90**	**23.24**	**6.365**	0.380	**1.302**
**SD**							

Sample Name (aniridia/48h-70mM)	Ct Mean Casp7	ct Mean TBP crt	ct Mean GUSB crt	Mean GUSB/TBP	ΔCt (Ct-Mean-GUSB/TE	ΔΔCt (Mean Ctrl - DCt)	Fold Change
AN-LFC15	30.203	22.286	21.867	22.08	8.13	-1.38	0.38
AN-LFC 5	30.602	24.533	22.916	23.72	6.88	-0.13	0.91
AN-LFC14	32.958	26.203	24.863	25.53	7.42	-0.68	0.62
AN-LFC16	32.353	25.501	23.790	24.65	7.71	-0.96	0.51
AN-LFC17	31.222	25.140	23.551	24.35	6.88	-0.13	0.91
AN-LFC 6	33.732	27.054	25.264	26.16	7.57	-0.83	0.56
AN-LFC 1	31.925	25.548	23.151	24.35	7.58	-0.83	0.56
AN-LFC 8	34.254	28.620	26.720	27.67	6.58	0.16	1.12
					7.343		0.661
**Mittelwert Aniridie**	**32.16**	**25.61**	**24.02**	**24.81**	**7.343**	-0.598	**0.661**
**SD**							

Casp8

Sample Name (ctr/48h-17.5mM)	Ct Mean Casp8	ct Mean TBP crt	ct Mean GUSB crt	Mean GUSB/TBP	ΔCt (Ct-Mean-GUSB/TE	ΔΔCt (Mean Ctrl - DCt)	Fold Change
LFC 153/P7	26.235	22.912	22.266	22.59	3.65	-0.09	0.94
LFC 396/P3	26.786	23.735	22.555	23.14	3.64	-0.08	0.95
LFC 416/P4	27.055	23.874	23.031	23.45	3.60	-0.04	0.97
LFC 427/P4	28.125	24.024	23.335	23.68	4.45	-0.88	0.54
LFC 428/P4	29.753	26.927	27.022	26.97	2.78	0.78	1.72
LFC 420/P4	26.570	23.703	22.296	23.00	3.57	-0.01	0.99
LFC 422/P4	26.646	23.732	23.083	23.41	3.24	0.32	1.25
					3.560	0.000	1.000
**Mittelwert crt**	**27.31**	**24.13**	**23.37**	**23.75**	**3.560**	0.000	**1.000**
Sample Name (ctr/48h-70mM)	Ct Mean Casp8	ct Mean TBP crt	ct Mean GUSB crt	Mean GUSB/TBP	ΔCt (Ct-Mean-GUSB/TE	ΔΔCt (Mean Ctrl - DCt)	Fold Change
LFC 491	29.871	25.515	23.809	24.66	5.21	-1.65	0.32
LFC 492	29.505	26.584	24.521	25.55	3.95	-0.39	0.76
LFC 777	29.673	26.439	24.614	25.53	4.15	-0.59	0.67
LFC 471	28.838	24.845	22.892	23.87	4.97	-1.41	0.38
LFC 467	28.296	24.522	23.640	24.08	4.21	-0.65	0.64
LFC 392	28.267	25.440	23.355	24.40	3.87	-0.31	0.81
LFC 486	30.079	27.170	25.655	26.41	3.67	-0.11	0.93
LFC 481	29.132	25.954	23.974	24.96	4.17	-0.61	0.66
					4.275		0.610
**Mittelwert**	**29.21**	**25.81**	**24.06**	**24.93**	**4.275**	-0.714	**0.610**
Sample Name (aniridia/48h-17.5mM)	Ct Mean Casp8	ct Mean TBP crt	ct Mean GUSB crt	Mean GUSB/TBP	ΔCt (Ct-Mean-GUSB/TE	ΔΔCt (Mean Ctrl - DCt)	Fold Change
AN-LFC12/P5	26.269	23.519	22.681	23.10	3.17	0.39	1.31
AN-LFC13/P5	25.814	22.550	22.107	22.33	3.49	0.07	1.05
AN-LFC6/P3	27.028	23.28	22.385	22.83	4.20	-0.64	0.64
AN-LFC 7/P3	26.298	22.556	22.025	22.29	4.01	-0.45	0.73
AN-LFC 8/P4	26.888	23.940	23.884	23.91	2.98	0.58	1.50
AN-LFC10/P4	28.168	23.739	24.482	24.11	4.06	-0.50	0.71
AN-LFC12/P5	27.601	23.508	22.997	23.25	4.35	-0.79	0.58
					3.749		0.878
**Mittelwert crt**	**26.87**	**23.30**	**22.94**	**23.12**	**3.749**	-0.188	**0.878**
**SD**							
Sample Name (aniridia/48h-70mM)	Ct Mean Casp8	ct Mean TBP crt	ct Mean GUSB crt	Mean GUSB/TBP	ΔCt (Ct-Mean-GUSB/TE	ΔΔCt (Mean Ctrl - DCt)	Fold Change
AN-LFC15	27.836	22.286	21.867	22.08	5.76	-2.20	0.22
AN-LFC 5	28.009	24.533	22.916	23.72	4.28	-0.72	0.61
AN-LFC14	30.147	26.203	24.863	25.53	4.61	-1.05	0.48
AN-LFC16	29.778	25.501	23.790	24.65	5.13	-1.57	0.34
AN-LFC17	28.769	25.140	23.551	24.35	4.42	-0.86	0.55
AN-LFC 6	30.522	27.054	25.264	26.16	4.36	-0.80	0.57
AN-LFC 1	29.685	25.548	23.151	24.35	5.33	-1.77	0.29
AN-LFC 8	31.836	28.620	26.720	27.67	4.17	-0.61	0.66
					4.760		0.435
**Mittelwert Aniridie**	**29.57**	**25.61**	**24.02**	**24.81**	**4.760**	-1.199	**0.435**
**SD**							

Casp9

Sample Name (ctr/48h-17.5mM)	Ct Mean Casp9	ct Mean TBP crt	ct Mean GUSB crt	Mean GUSB/TBP	ΔCt (Ct-Mean-GUSB/TE	ΔΔCt (Mean Ctrl - DCt)	Fold Change
LFC 153/P7	24.736	22.912	22.266	22.59	2.15	-0.07	0.95
LFC 396/P3	25.634	23.735	22.555	23.14	2.49	-0.41	0.75
LFC 416/P4	25.286	23.874	23.031	23.45	1.83	0.24	1.18
LFC 427/P4	25.147	24.024	23.335	23.68	1.47	0.61	1.52
LFC 428/P4	28.779	26.927	27.022	26.97	1.80	0.27	1.21
LFC 420/P4	25.580	23.703	22.296	23.00	2.58	-0.51	0.70
LFC 422/P4	25.609	23.732	23.083	23.41	2.20	-0.13	0.92
					2.075	0.000	1.000
**Mittelwert crt**	**25.82**	**24.13**	**23.37**	**23.75**	**2.075**	0.000	**1.000**

Sample Name (ctr/48h-70mM)	Ct Mean Casp9	ct Mean TBP crt	ct Mean GUSB crt	Mean GUSB/TBP	ΔCt (Ct-Mean-GUSB/TE	ΔΔCt (Mean Ctrl - DCt)	Fold Change
LFC 491	28.151	25.515	23.809	24.66	3.49	-1.41	0.38
LFC 492	27.460	26.584	24.521	25.55	1.91	0.17	1.12
LFC 777	27.733	26.439	24.614	25.53	2.21	-0.13	0.91
LFC 471	27.134	24.845	22.892	23.87	3.27	-1.19	0.44
LFC 467	26.283	24.522	23.640	24.08	2.20	-0.13	0.92
LFC 392	26.099	25.440	23.355	24.40	1.70	0.37	1.30
LFC 486	28.474	27.170	25.655	26.41	2.06	0.01	1.01
LFC 481	26.899	25.954	23.974	24.96	1.94	0.14	1.10
					2.346		0.829
**Mittelwert**	**27.28**	**25.81**	**24.06**	**24.93**	**2.346**	-0.271	**0.829**

Sample Name (aniridia/48h-17.5mM)	Ct Mean Casp9	ct Mean TBP crt	ct Mean GUSB crt	Mean GUSB/TBP	ΔCt (Ct-Mean-GUSB/TE	ΔΔCt (Mean Ctrl - DCt)	Fold Change
AN-LFC12/P5	25.015	23.519	22.681	23.10	1.92	0.16	1.12
AN-LFC13/P5	24.810	22.550	22.107	22.33	2.48	-0.41	0.75
AN-LFC6/P3	24.739	23.28	22.385	22.83	1.91	0.17	1.12
AN-LFC 7/P3	24.345	22.556	22.025	22.29	2.05	0.02	1.01
AN-LFC 8/P4	25.802	23.940	23.884	23.91	1.89	0.18	1.14
AN-LFC10/P4	25.775	23.739	24.482	24.11	1.66	0.41	1.33
AN-LFC12/P5	26.046	23.508	22.997	23.25	2.79	-0.72	0.61
					2.101		0.982
**Mittelwert crt**	**25.22**	**23.30**	**22.94**	**23.12**	**2.101**	-0.026	**0.982**
**SD**							

Sample Name (aniridia/48h-70mM)	Ct Mean Casp9	ct Mean TBP crt	ct Mean GUSB crt	Mean GUSB/TBP	ΔCt (Ct-Mean-GUSB/TE	ΔΔCt (Mean Ctrl - DCt)	Fold Change
AN-LFC15	25.778	22.286	21.867	22.08	3.70	-1.63	0.32
AN-LFC 5	26.243	24.533	22.916	23.72	2.52	-0.44	0.73
AN-LFC14	28.009	26.203	24.863	25.53	2.48	-0.40	0.76
AN-LFC16	27.767	25.501	23.790	24.65	3.12	-1.05	0.48
AN-LFC17	26.141	25.140	23.551	24.35	1.80	0.28	1.21
AN-LFC 6	27.915	27.054	25.264	26.16	1.76	0.32	1.25
AN-LFC 1	26.401	25.548	23.151	24.35	2.05	0.02	1.02
AN-LFC 8	29.404	28.620	26.720	27.67	1.73	0.34	1.27
					2.394		0.801
**Mittelwert Aniridie**	**27.21**	**25.61**	**24.02**	**24.81**	**2.394**	-0.320	**0.801**
**SD**							

Casp10

Sample Name (ctr/48h-17.5mM)	Ct Mean Casp10	ct Mean TBP crt	ct Mean GUSB crt	Mean GUSB/TBP	ΔCt (Ct-Mean-GUSB/TE	ΔΔCt (Mean Ctrl - DCt)	Fold Change
LFC 153/P7	25.980	22.912	22.266	22.59	3.39	-0.18	0.88
LFC 396/P3	25.749	23.735	22.555	23.14	2.60	0.60	1.52
LFC 416/P4	25.855	23.874	23.031	23.45	2.40	0.80	1.75
LFC 427/P4	27.504	24.024	23.335	23.68	3.82	-0.62	0.65
LFC 428/P4	29.618	26.927	27.022	26.97	2.64	0.56	1.48
LFC 420/P4	27.936	23.703	22.296	23.00	4.94	-1.73	0.30
LFC 422/P4	26.058	23.732	23.083	23.41	2.65	0.56	1.47
					3.207	0.000	1.000
**Mittelwert crt**	**26.96**	**24.13**	**23.37**	**23.75**	**3.207**	0.000	**1.000**

Sample Name (ctr/48h-70mM)	Ct Mean Casp10	ct Mean TBP crt	ct Mean GUSB crt	Mean GUSB/TBP	ΔCt (Ct-Mean-GUSB/TE	ΔΔCt (Mean Ctrl - DCt)	Fold Change
LFC 491	30.948	25.515	23.809	24.66	6.29	-3.08	0.12
LFC 492	31.161	26.584	24.521	25.55	5.61	-2.40	0.19
LFC 777	31.749	26.439	24.614	25.53	6.22	-3.02	0.12
LFC 471	30.709	24.845	22.892	23.87	6.84	-3.63	0.08
LFC 467	29.700	24.522	23.640	24.08	5.62	-2.41	0.19
LFC 392	29.480	25.440	23.355	24.40	5.08	-1.88	0.27
LFC 486	31.989	27.170	25.655	26.41	5.58	-2.37	0.19
LFC 481	30.041	25.954	23.974	24.96	5.08	-1.87	0.27
					5.789		0.167
**Mittelwert**	**30.72**	**25.81**	**24.06**	**24.93**	**5.789**	-2.582	**0.167**

Sample Name (aniridia/48h-17.5mM)	Ct Mean Casp10	ct Mean TBP crt	ct Mean GUSB crt	Mean GUSB/TBP	ΔCt (Ct-Mean-GUSB/TE	ΔΔCt (Mean Ctrl - DCt)	Fold Change
AN-LFC12/P5	26.056	23.519	22.681	23.10	2.96	0.25	1.19
AN-LFC13/P5	26.001	22.550	22.107	22.33	3.67	-0.46	0.72
AN-LFC6/P3	25.89	23.28	22.385	22.83	3.06	0.15	1.11
AN-LFC 7/P3	25.895	22.556	22.025	22.29	3.60	-0.40	0.76
AN-LFC 8/P4	26.534	23.940	23.884	23.91	2.62	0.59	1.50
AN-LFC10/P4	26.753	23.739	24.482	24.11	2.64	0.56	1.48
AN-LFC12/P5	28.054	23.508	22.997	23.25	4.80	-1.59	0.33
					3.337		0.914
**Mittelwert crt**	**26.45**	**23.30**	**22.94**	**23.12**	**3.337**	-0.129	**0.914**
**SD**							

Sample Name (aniridia/48h-70mM)	Ct Mean Casp10	ct Mean TBP crt	ct Mean GUSB crt	Mean GUSB/TBP	ΔCt (Ct-Mean-GUSB/TE	ΔΔCt (Mean Ctrl - DCt)	Fold Change
AN-LFC15	27.268	22.286	21.867	22.08	5.19	-1.98	0.25
AN-LFC 5	28.716	24.533	22.916	23.72	4.99	-1.78	0.29
AN-LFC14	30.679	26.203	24.863	25.53	5.15	-1.94	0.26
AN-LFC16	29.922	25.501	23.790	24.65	5.28	-2.07	0.24
AN-LFC17	30.241	25.140	23.551	24.35	5.90	-2.69	0.16
AN-LFC 6	32.120	27.054	25.264	26.16	5.96	-2.75	0.15
AN-LFC 1	30.219	25.548	23.151	24.35	5.87	-2.66	0.16
AN-LFC 8	32.357	28.620	26.720	27.67	4.69	-1.48	0.36
					5.377		0.222
**Mittelwert Aniridie**	**30.19**	**25.61**	**24.02**	**24.81**	**5.377**	-2.170	**0.222**
**SD**							

BAX

**Sample Name (ctr/48h-17.5mM)**	Ct Mean BAX	ct Mean TBP crt	ct Mean GUSB crt	Mean GUSB/TBP	ΔCt (Ct-Mean-GUSB/TE	ΔΔCt (Mean Ctrl - DCt)	Fold Change
LFC 153/P7	20.971	22.912	22.266	22.59	-1.62	-0.30	0.82
LFC 396/P3	21.137	23.735	22.555	23.14	-2.01	0.10	1.07
LFC 416/P4	21.78	23.874	23.031	23.45	-1.67	-0.24	0.85
LFC 427/P4	21.319	24.024	23.335	23.68	-2.36	0.45	1.36
LFC 428/P4	24.789	26.927	27.022	26.97	-2.19	0.27	1.21
LFC 420/P4	21.398	23.703	22.296	23.00	-1.60	-0.31	0.81
LFC 422/P4	21.465	23.732	23.083	23.41	-1.94	0.03	1.02
					-1.913	0.000	1.000
**Mittelwert crt**	**21.84**	**24.13**	**23.37**	**23.75**	**-1.913**	0.000	**1.000**

**Sample Name (ctr/48h-70mM)**	Ct Mean BAX	ct Mean TBP crt	ct Mean GUSB crt	Mean GUSB/TBP	ΔCt (Ct-Mean-GUSB/TE	ΔΔCt (Mean Ctrl - DCt)	Fold Change
LFC 491	21.951	25.515	23.809	24.66	-2.71	0.80	1.74
LFC 492	23.149	26.584	24.521	25.55	-2.40	0.49	1.41
LFC 777	22.849	26.439	24.614	25.53	-2.68	0.77	1.70
LFC 471	22.510	24.845	22.892	23.87	-1.36	-0.55	0.68
LFC 467	21.668	24.522	23.640	24.08	-2.41	0.50	1.41
LFC 392	21.966	25.440	23.355	24.40	-2.43	0.52	1.43
LFC 486	24.543	27.170	25.655	26.41	-1.87	-0.04	0.97
LFC 481	22.730	25.954	23.974	24.96	-2.23	0.32	1.25
					-2.262		1.274
**Mittelwert**	**22.67**	**25.81**	**24.06**	**24.93**	**-2.262**	0.350	**1.274**

**Sample Name (aniridia/48h-17.5mM)**	Ct Mean BAX	ct Mean TBP crt	ct Mean GUSB crt	Mean GUSB/TBP	ΔCt (Ct-Mean-GUSB/TE	ΔΔCt (Mean Ctrl - DCt)	Fold Change
AN-LFC13/P5	20.270	22.550	22.107	22.33	-2.06	0.15	1.11
AN-LFC 5/P4	23.942	25.567	22.614	24.09	-0.15	-1.76	0.29
AN-LFC6/P3	20.977	23.28	22.385	22.83	-1.86	-0.06	0.96
AN-LFC 7/P3	20.881	22.556	22.025	22.29	-1.41	-0.50	0.71
AN-LFC 8/P4	22.633	23.940	23.884	23.91	-1.28	-0.63	0.64
AN-LFC10/P4	22.778	23.739	24.482	24.11	-1.33	-0.58	0.67
AN-LFC12/P5	21.859	23.508	22.997	23.25	-1.39	-0.52	0.70
					-1.354		0.679
**Mittelwert crt**	**21.91**	**23.59**	**22.93**	**23.26**	**-1.354**	-0.559	**0.679**
**SD**							

**Sample Name (aniridia/48h-70mM)**	Ct Mean BAX	ct Mean TBP crt	ct Mean GUSB crt	Mean GUSB/TBP	ΔCt (Ct-Mean-GUSB/TE	ΔΔCt (Mean Ctrl - DCt)	Fold Change
AN-LFC15	20.793	22.286	21.867	22.08	-1.28	-0.63	0.65
AN-LFC 5	21.899	24.533	22.916	23.72	-1.82	-0.09	0.94
AN-LFC14	23.997	26.203	24.863	25.53	-1.54	-0.38	0.77
AN-LFC16	23.012	25.501	23.790	24.65	-1.63	-0.28	0.82
AN-LFC17	22.060	25.140	23.551	24.35	-2.29	0.37	1.30
AN-LFC 6	24.152	27.054	25.264	26.16	-2.01	0.09	1.07
AN-LFC 1	22.868	25.548	23.151	24.35	-1.48	-0.43	0.74
AN-LFC 8	25.710	28.620	26.720	27.67	-1.96	0.05	1.03
					-1.751		0.894
**Mittelwert Aniridie**	**23.06**	**25.61**	**24.02**	**24.81**	**-1.751**	-0.161	**0.894**
**SD**							

BID

Sample Name (ctr/48h-17.5mM)	Ct Mean BID	ct Mean TBP crt	ct Mean GUSB crt	Mean GUSB/TBP	ΔCt (Ct-Mean-GUSB/TE	ΔΔCt (Mean Ctrl - DCt)	Fold Change
LFC 153/P7	22.452	22.912	22.266	22.59	-0.14	-1.55	0.34
LFC 396/P3	21.999	23.735	22.555	23.14	-1.15	-0.54	0.69
LFC 416/P4	21.672	23.874	23.031	23.45	-1.78	0.09	1.07
LFC 427/P4	21.538	24.024	23.335	23.68	-2.14	0.45	1.37
LFC 428/P4	24.721	26.927	27.022	26.97	-2.25	0.56	1.48
LFC 420/P4	20.704	23.703	22.296	23.00	-2.30	0.61	1.52
LFC 422/P4	21.337	23.732	23.083	23.41	-2.07	0.38	1.30
					-1.689	0.000	1.000
**Mittelwert crt**	**22.06**	**24.13**	**23.37**	**23.75**	**-1.689**	0.000	**1.000**

Sample Name (ctr/48h-70mM)	Ct Mean BID	ct Mean TBP crt	ct Mean GUSB crt	Mean GUSB/TBP	ΔCt (Ct-Mean-GUSB/TE	ΔΔCt (Mean Ctrl - DCt)	Fold Change
LFC 491	23.393	25.515	23.809	24.66	-1.27	-0.42	0.75
LFC 492	23.445	26.584	24.521	25.55	-2.11	0.42	1.34
LFC 777	23.038	26.439	24.614	25.53	-2.49	0.80	1.74
LFC 471	21.760	24.845	22.892	23.87	-2.11	0.42	1.34
LFC 467	21.833	24.522	23.640	24.08	-2.25	0.56	1.47
LFC 392	22.608	25.440	23.355	24.40	-1.79	0.10	1.07
LFC 486	24.192	27.170	25.655	26.41	-2.22	0.53	1.45
LFC 481	22.761	25.954	23.974	24.96	-2.20	0.51	1.43
					-2.054		1.288
**Mittelwert**	**22.88**	**25.81**	**24.06**	**24.93**	**-2.054**	0.365	**1.288**

Sample Name (aniridia/48h-17.5mM)	Ct Mean BID	ct Mean TBP crt	ct Mean GUSB crt	Mean GUSB/TBP	ΔCt (Ct-Mean-GUSB/TE	ΔΔCt (Mean Ctrl - DCt)	Fold Change
AN-LFC12/P5	20.832	23.519	22.681	23.10	-2.27	0.58	1.49
AN-LFC13/P5	21.910	22.550	22.107	22.33	-0.42	-1.27	0.41
AN-LFC 5/P4	20.653	25.567	22.614	24.09	-3.44	1.75	3.36
AN-LFC6/P3	21.759	23.28	22.385	22.83	-1.07	-0.62	0.65
AN-LFC 7/P3	20.243	22.556	22.025	22.29	-2.05	0.36	1.28
AN-LFC 8/P4	21.582	23.940	23.884	23.91	-2.33	0.64	1.56
AN-LFC10/P4	21.712	23.739	24.482	24.11	-2.40	0.71	1.64
AN-LFC12/P5	20.685	23.508	22.997	23.25	-2.57	0.88	1.84
					-2.067		1.300
**Mittelwert crt**	**21.17**	**23.58**	**22.90**	**23.24**	**-2.067**	0.378	**1.300**
**SD**							

Sample Name (aniridia/48h-70mM)	Ct Mean BID	ct Mean TBP crt	ct Mean GUSB crt	Mean GUSB/TBP	ΔCt (Ct-Mean-GUSB/TE	ΔΔCt (Mean Ctrl - DCt)	Fold Change
AN-LFC15	21.015	22.286	21.867	22.08	-1.06	-0.63	0.65
AN-LFC 5	22.369	24.533	22.916	23.72	-1.36	-0.33	0.79
AN-LFC14	24.161	26.203	24.863	25.53	-1.37	-0.32	0.80
AN-LFC16	22.716	25.501	23.790	24.65	-1.93	0.24	1.18
AN-LFC17	22.510	25.140	23.551	24.35	-1.84	0.15	1.11
AN-LFC 6	23.984	27.054	25.264	26.16	-2.17	0.49	1.40
AN-LFC 1	22.908	25.548	23.151	24.35	-1.44	-0.25	0.84
AN-LFC 8	25.211	28.620	26.720	27.67	-2.46	0.77	1.71
					-1.704		1.010
**Mittelwert Aniridie**	**23.11**	**25.61**	**24.02**	**24.81**	**-1.704**	0.014	**1.010**
**SD**							

Bcl2

Sample Name (ctr/48h-17.5mM)	Ct Mean Bcl2	ct Mean TBP crt	ct Mean GUSB crt	Mean GUSB/TBP	ΔCt (Ct-Mean-GUSB/TE	ΔΔCt (Mean Ctrl - DCt)	Fold Change
LFC 153/P7	28.606	22.912	22.266	22.59	6.02	-1.09	0.47
LFC 396/P3	27.224	23.735	22.555	23.14	4.08	0.85	1.80
LFC 416/P4	28.109	23.874	23.031	23.45	4.66	0.27	1.21
LFC 427/P4	28.789	24.024	23.335	23.68	5.11	-0.18	0.88
LFC 428/P4	32.184	26.927	27.022	26.97	5.21	-0.28	0.82
LFC 420/P4	28.143	23.703	22.296	23.00	5.14	-0.22	0.86
LFC 422/P4	27.680	23.732	23.083	23.41	4.27	0.65	1.57
					4.927	0.000	1.000
**Mittelwert crt**	**28.68**	**24.13**	**23.37**	**23.75**	**4.927**	0.000	**1.000**

Sample Name (ctr/48h-70mM)	Ct Mean Bcl2	ct Mean TBP crt	ct Mean GUSB crt	Mean GUSB/TBP	ΔCt (Ct-Mean-GUSB/TE	ΔΔCt (Mean Ctrl - DCt)	Fold Change
LFC 491	31.835	25.515	23.809	24.66	7.17	-2.25	0.21
LFC 492	31.503	26.584	24.521	25.55	5.95	-1.02	0.49
LFC 777	30.957	26.439	24.614	25.53	5.43	-0.50	0.71
LFC 471	31.196	24.845	22.892	23.87	7.33	-2.40	0.19
LFC 467	29.957	24.522	23.640	24.08	5.88	-0.95	0.52
LFC 392	29.675	25.440	23.355	24.40	5.28	-0.35	0.78
LFC 486	32.565	27.170	25.655	26.41	6.15	-1.23	0.43
LFC 481	29.545	25.954	23.974	24.96	4.58	0.35	1.27
					5.971		0.485
**Mittelwert**	**30.90**	**25.81**	**24.06**	**24.93**	**5.971**	-1.044	**0.485**

Sample Name (aniridia/48h-17.5mM)	Ct Mean Bcl2	ct Mean TBP crt	ct Mean GUSB crt	Mean GUSB/TBP	ΔCt (Ct-Mean-GUSB/TE	ΔΔCt (Mean Ctrl - DCt)	Fold Change
AN-LFC12/P5	27.321	23.519	22.681	23.10	4.22	0.71	1.63
AN-LFC13/P5	27.843	22.550	22.107	22.33	5.51	-0.59	0.67
AN-LFC 5/P4	30.528	25.567	22.614	24.09	6.44	-1.51	0.35
AN-LFC6/P3	27.897	23.28	22.385	22.83	5.06	-0.14	0.91
AN-LFC 7/P3	27.050	22.556	22.025	22.29	4.76	0.17	1.12
AN-LFC 8/P4	28.628	23.940	23.884	23.91	4.72	0.21	1.16
AN-LFC12/P5	28.322	23.508	22.997	23.25	5.07	-0.14	0.91
					5.112		0.880
**Mittelwert crt**	**28.23**	**23.56**	**22.67**	**23.11**	**5.112**	-0.185	**0.880**
**SD**							

Sample Name (aniridia/48h-70mM)	Ct Mean Bcl2	ct Mean TBP crt	ct Mean GUSB crt	Mean GUSB/TBP	ΔCt (Ct-Mean-GUSB/TE	ΔΔCt (Mean Ctrl - DCt)	Fold Change
AN-LFC15	27.352	22.286	21.867	22.08	5.28	-0.35	0.79
AN-LFC 5	28.866	24.533	22.916	23.72	5.14	-0.21	0.86
AN-LFC14	30.621	26.203	24.863	25.53	5.09	-0.16	0.89
AN-LFC16	30.168	25.501	23.790	24.65	5.52	-0.60	0.66
AN-LFC17	28.781	25.140	23.551	24.35	4.44	0.49	1.41
AN-LFC 6	30.209	27.054	25.264	26.16	4.05	0.88	1.84
AN-LFC 1	29.648	25.548	23.151	24.35	5.30	-0.37	0.77
AN-LFC 8	31.919	28.620	26.720	27.67	4.25	0.68	1.60
					4.883		1.031
**Mittelwert Aniridie**	**29.70**	**25.61**	**24.02**	**24.81**	**4.883**	0.044	**1.031**
**SD**							

BIRC5

Sample Name (ctr/48h-17.5mM)	Ct Mean BIRC5	ct Mean TBP crt	ct Mean GUSB crt	Mean GUSB/TBP	ΔCt (Ct-Mean-GUSB/TE	ΔΔCt (Mean Ctrl - DCt)	Fold Change
LFC 153/P7	22.383	22.912	22.266	22.59	-0.21	0.86	1.82
LFC 396/P3	23.218	23.735	22.555	23.14	0.07	0.58	1.50
LFC 416/P4	24.938	23.874	23.031	23.45	1.49	-0.83	0.56
LFC 427/P4	22.832	24.024	23.335	23.68	-0.85	1.50	2.83
LFC 428/P4	29.727	26.927	27.022	26.97	2.75	-2.10	0.23
LFC 420/P4	23.994	23.703	22.296	23.00	0.99	-0.34	0.79
LFC 422/P4	23.744	23.732	23.083	23.41	0.34	0.32	1.25
					0.656	0.000	1.000
**Mittelwert crt**	**24.41**	**24.13**	**23.37**	**23.75**	**0.656**	0.000	**1.000**

Sample Name (ctr/48h-70mM)	Ct Mean BIRC5	ct Mean TBP crt	ct Mean GUSB crt	Mean GUSB/TBP	ΔCt (Ct-Mean-GUSB/TE	ΔΔCt (Mean Ctrl - DCt)	Fold Change
LFC 491	23.310	25.515	23.809	24.66	-1.35	2.01	4.02
LFC 492	23.241	26.584	24.521	25.55	-2.31	2.97	7.82
LFC 471	22.497	24.845	22.892	23.87	-1.37	2.03	4.08
LFC 467	22.175	24.522	23.640	24.08	-1.91	2.56	5.90
LFC 392	22.122	25.440	23.355	24.40	-2.28	2.93	7.63
LFC 486	24.755	27.170	25.655	26.41	-1.66	2.31	4.97
LFC 481	22.902	25.954	23.974	24.96	-2.06	2.72	6.57
					-1.848		5.670
**Mittelwert**	**23.00**	**25.72**	**23.98**	**24.85**	**-1.848**	2.503	**5.670**

Sample Name (aniridia/48h-17.5mM)	Ct Mean BIRC5	ct Mean TBP crt	ct Mean GUSB crt	Mean GUSB/TBP	ΔCt (Ct-Mean-GUSB/TE	ΔΔCt (Mean Ctrl - DCt)	Fold Change
AN-LFC12/P5	22.434	23.519	22.681	23.10	-0.67	1.32	2.50
AN-LFC13/P5	22.005	22.550	22.107	22.33	-0.32	0.98	1.97
AN-LFC 5/P4	25.292	25.567	22.614	24.09	1.20	-0.55	0.68
AN-LFC6/P3	22.623	23.28	22.385	22.83	-0.21	0.87	1.82
AN-LFC 7/P3	22.171	22.556	22.025	22.29	-0.12	0.78	1.71
AN-LFC 8/P4	24.130	23.940	23.884	23.91	0.22	0.44	1.35
AN-LFC10/P4	23.031	23.739	24.482	24.11	-1.08	1.74	3.33
AN-LFC12/P5	22.919	23.508	22.997	23.25	-0.33	0.99	1.98
					-0.164		1.765
**Mittelwert crt**	**23.08**	**23.58**	**22.90**	**23.24**	**-0.164**	0.819	**1.765**
**SD**							

Sample Name (aniridia/48h-70mM)	Ct Mean BIRC5	ct Mean TBP crt	ct Mean GUSB crt	Mean GUSB/TBP	ΔCt (Ct-Mean-GUSB/TE	ΔΔCt (Mean Ctrl - DCt)	Fold Change
AN-LFC15	23.459	22.286	21.867	22.08	1.38	-0.73	0.60
AN-LFC 5	22.043	24.533	22.916	23.72	-1.68	2.34	5.05
AN-LFC14	23.425	26.203	24.863	25.53	-2.11	2.76	6.79
AN-LFC16	22.940	25.501	23.790	24.65	-1.71	2.36	5.14
AN-LFC17	21.688	25.140	23.551	24.35	-2.66	3.31	9.94
AN-LFC 6	23.103	27.054	25.264	26.16	-3.06	3.71	13.10
AN-LFC 1	21.521	25.548	23.151	24.35	-2.83	3.48	11.19
AN-LFC 8	24.376	28.620	26.720	27.67	-3.29	3.95	15.45
					-1.994		6.273
**Mittelwert Aniridie**	**22.82**	**25.61**	**24.02**	**24.81**	**-1.994**	2.649	**6.273**
**SD**							

XIAP

Sample Name (ctr/48h-17.5mM)	Ct Mean XIAP	ct Mean TBP crt	ct Mean GUSB crt	Mean GUSB/TBP	ΔCt (Ct-Mean-GUSB/TE	ΔΔCt (Mean Ctrl - DCt)	Fold Change
LFC 153/P7	26.129	22.912	22.266	22.59	3.54	-0.05	0.97
LFC 396/P3	26.723	23.735	22.555	23.14	3.58	-0.08	0.94
LFC 416/P4	26.648	23.874	23.031	23.45	3.20	0.30	1.23
LFC 427/P4	26.796	24.024	23.335	23.68	3.12	0.38	1.30
LFC 420/P4	26.949	23.703	22.296	23.00	3.95	-0.45	0.73
LFC 422/P4	26.995	23.732	23.083	23.41	3.59	-0.09	0.94
					3.495	0.000	1.000
**Mittelwert crt**	**26.71**	**23.66**	**22.76**	**23.21**	**3.495**	0.000	**1.000**

Sample Name (ctr/48h-70mM)	Ct Mean XIAP	ct Mean TBP crt	ct Mean GUSB crt	Mean GUSB/TBP	ΔCt (Ct-Mean-GUSB/TE	ΔΔCt (Mean Ctrl - DCt)	Fold Change
LFC 491	26.777	25.515	23.809	24.66	2.12	1.38	2.60
LFC 492	26.906	26.584	24.521	25.55	1.35	2.14	4.41
LFC 777	26.832	26.439	24.614	25.53	1.31	2.19	4.56
LFC 471	27.116	24.845	22.892	23.87	3.25	0.25	1.19
LFC 467	27.186	24.522	23.640	24.08	3.11	0.39	1.31
LFC 392	27.225	25.440	23.355	24.40	2.83	0.67	1.59
LFC 486	27.583	27.170	25.655	26.41	1.17	2.32	5.01
LFC 481	26.924	25.954	23.974	24.96	1.96	1.53	2.90
					2.136		2.565
**Mittelwert**	**27.07**	**25.81**	**24.06**	**24.93**	**2.136**	1.359	**2.565**

Sample Name (aniridia/48h-17.5mM)	Ct Mean XIAP	ct Mean TBP crt	ct Mean GUSB crt	Mean GUSB/TBP	ΔCt (Ct-Mean-GUSB/TE	ΔΔCt (Mean Ctrl - DCt)	Fold Change
AN-LFC12/P5	26.624	23.519	22.681	23.10	3.52	-0.03	0.98
AN-LFC13/P5	25.945	22.550	22.107	22.33	3.62	-0.12	0.92
AN-LFC 5/P4	27.113	25.567	22.614	24.09	3.02	0.47	1.39
AN-LFC6/P3	26.508	23.28	22.385	22.83	3.68	-0.18	0.88
AN-LFC 7/P3	26.682	22.556	22.025	22.29	4.39	-0.90	0.54
AN-LFC 8/P4	26.238	23.940	23.884	23.91	2.33	1.17	2.25
AN-LFC10/P4	26.797	23.739	24.482	24.11	2.69	0.81	1.75
AN-LFC12/P5	26.787	23.508	22.997	23.25	3.54	-0.04	0.97
					3.348		1.107
**Mittelwert crt**	**26.59**	**23.58**	**22.90**	**23.24**	**3.348**	0.147	**1.107**
**SD**							

Sample Name (aniridia/48h-70mM)	Ct Mean XIAP	ct Mean TBP crt	ct Mean GUSB crt	Mean GUSB/TBP	ΔCt (Ct-Mean-GUSB/TE	ΔΔCt (Mean Ctrl - DCt)	Fold Change
AN-LFC15	25.694	22.286	21.867	22.08	3.62	-0.12	0.92
AN-LFC 5	26.219	24.533	22.916	23.72	2.50	1.00	2.00
AN-LFC14	27.368	26.203	24.863	25.53	1.84	1.66	3.16
AN-LFC16	27.132	25.501	23.790	24.65	2.49	1.01	2.01
AN-LFC17	26.618	25.140	23.551	24.35	2.27	1.22	2.33
AN-LFC 6	27.581	27.054	25.264	26.16	1.42	2.07	4.21
AN-LFC 1	26.563	25.548	23.151	24.35	2.21	1.28	2.43
AN-LFC 8	28.456	28.620	26.720	27.67	0.79	2.71	6.53
					2.141		2.555
**Mittelwert Aniridie**	**26.95**	**25.61**	**24.02**	**24.81**	**2.141**	1.354	**2.555**
**SD**							

CDKN1B(p27)

Sample Name (ctr/48h-17.5mM)	Ct Mean CDKN1B	ct Mean TBP crt	ct Mean GUSB crt	Mean GUSB/TBP	ΔCt (Ct-Mean-GUSB/TE	ΔΔCt (Mean Ctrl - DCt)	Fold Change
LFC 153/P7	24.687	22.912	22.266	22.59	2.10	0.16	1.12
LFC 396/P3	25.217	23.735	22.555	23.14	2.07	0.19	1.14
LFC 416/P4	25.458	23.874	23.031	23.45	2.01	0.25	1.19
LFC 427/P4	26.434	24.024	23.335	23.68	2.75	-0.50	0.71
LFC 428/P4	28.953	26.927	27.022	26.97	1.98	0.28	1.21
LFC 420/P4	25.165	23.703	22.296	23.00	2.17	0.09	1.07
LFC 422/P4	26.149	23.732	23.083	23.41	2.74	-0.48	0.72
					2.259	0.000	1.000
**Mittelwert crt**	**26.01**	**24.13**	**23.37**	**23.75**	**2.259**	0.000	**1.000**

Sample Name (ctr/48h-70mM)	Ct Mean CDKN1B	ct Mean TBP crt	ct Mean GUSB crt	Mean GUSB/TBP	ΔCt (Ct-Mean-GUSB/TE	ΔΔCt (Mean Ctrl - DCt)	Fold Change
LFC 491	27.788	25.515	23.809	24.66	3.13	-0.87	0.55
LFC 492	28.275	26.584	24.521	25.55	2.72	-0.46	0.73
LFC 777	27.969	26.439	24.614	25.53	2.44	-0.18	0.88
LFC 471	26.945	24.845	22.892	23.87	3.08	-0.82	0.57
LFC 467	27.788	24.522	23.640	24.08	3.71	-1.45	0.37
LFC 392	28.275	25.440	23.355	24.40	3.88	-1.62	0.33
LFC 486	27.969	27.170	25.655	26.41	1.56	0.70	1.63
LFC 481	26.945	25.954	23.974	24.96	1.98	0.28	1.21
					2.811		0.682
**Mittelwert**	**27.74**	**25.81**	**24.06**	**24.93**	**2.811**	-0.552	**0.682**

Sample Name (aniridia/48h-17.5mM)	Ct Mean CDKN1B	ct Mean TBP crt	ct Mean GUSB crt	Mean GUSB/TBP	ΔCt (Ct-Mean-GUSB/TE	ΔΔCt (Mean Ctrl - DCt)	Fold Change
AN-LFC12/P5	25.461	23.519	22.681	23.10	2.36	-0.10	0.93
AN-LFC13/P5	23.893	22.550	22.107	22.33	1.56	0.69	1.62
AN-LFC 5/P4	26.945	25.567	22.614	24.09	2.85	-0.60	0.66
AN-LFC6/P3	24.595	23.28	22.385	22.83	1.76	0.50	1.41
AN-LFC 7/P3	24.863	22.556	22.025	22.29	2.57	-0.31	0.80
AN-LFC 8/P4	25.727	23.940	23.884	23.91	1.82	0.44	1.36
AN-LFC10/P4	25.393	23.739	24.482	24.11	1.28	0.98	1.97
AN-LFC12/P5	24.930	23.508	22.997	23.25	1.68	0.58	1.50
					1.987		1.208
**Mittelwert crt**	**25.23**	**23.58**	**22.90**	**23.24**	**1.987**	0.273	**1.208**
**SD**							

Sample Name (aniridia/48h-70mM)	Ct Mean CDKN1B	ct Mean TBP crt	ct Mean GUSB crt	Mean GUSB/TBP	ΔCt (Ct-Mean-GUSB/TE	ΔΔCt (Mean Ctrl - DCt)	Fold Change
AN-LFC15	25.293	22.286	21.867	22.08	3.22	-0.96	0.52
AN-LFC 5	27.175	24.533	22.916	23.72	3.45	-1.19	0.44
AN-LFC14	28.116	26.203	24.863	25.53	2.58	-0.32	0.80
AN-LFC16	27.073	25.501	23.790	24.65	2.43	-0.17	0.89
AN-LFC17	25.293	25.140	23.551	24.35	0.95	1.31	2.48
AN-LFC 6	27.175	27.054	25.264	26.16	1.02	1.24	2.37
AN-LFC 1	28.116	25.548	23.151	24.35	3.77	-1.51	0.35
					2.487		0.854
**Mittelwert Aniridie**	**26.89**	**25.18**	**23.63**	**24.40**	**2.487**	-0.227	**0.854**
**SD**							

B515LFC	control-LFC	17.5mM FITC	17.5mM BAX	70mM FITC	70mM BAX
	geomean	14595.4	32612.6	10294.1	26629.2
			2.234443729		2.586841006
AN2	aniridia-LFC	17.5mM FITC	17.5mM BAX	70mM FITC	70mM BAX
	geomean	16187.7	30325.2	13404.9	28400
			1.873348283		2.118628263
B550LFC	control-LFC	17.5mM FITC	17.5mM BAX	70mM FITC	70mM BAX
	geomean	10938.1	16640.7	12750.2	19473.8
			1.521351972		1.527332905
AN5	aniridia-LFC	17.5mM FITC	17.5mM BAX	70mM FITC	70mM BAX
	geomean	30416.9	38443.2	21914.4	28041.2
			1.263876332		1.279578724
1748LFC	control-LFC	17.5mM FITC	17.5mM BAX	70mM FITC	70mM BAX
	geomean	20616.7	40846.7	13595.6	21181.6
			1.981243361		1.557974639
AN14	aniridia-LFC	17.5mM FITC	17.5mM BAX	70mM FITC	70mM BAX
	geomean	26102.7	35553.7	19158.6	21391.8
			1.362069824		1.116563841

B515LFC	control-LFC	17.5mM FITC	17.5mM Bcl2	70mM FITC	70mM Bcl2
	geomean	14595.4	180973.9	10294.1	148791.3
			12.39937926		14.45403678
AN2	aniridia-LFC	17.5mM FITC	17.5mM Bcl2	70mM FITC	70mM Bcl2
	geomean	16187.7	164525.3	13404.9	228276.7
			10.16359952		17.02934748
B550LFC	control-LFC	17.5mM FITC	17.5mM Bcl2	70mM FITC	70mM Bcl2
	geomean	10938.1	152238.6	12750.2	225657.2
			13.9181942		17.6983263
AN5	aniridia-LFC	17.5mM FITC	17.5mM Bcl2	70mM FITC	70mM Bcl2
	geomean	30416.9	366451.6	21914.4	345715.4
			12.04763142		15.77571825
1748LFC	control-LFC	17.5mM FITC	17.5mM Bcl2	70mM FITC	70mM Bcl2
	geomean	20616.7	220210.4	13595.6	184782.1
			10.68116624		13.59131631
AN14	aniridia-LFC	17.5mM FITC	17.5mM Bcl2	70mM FITC	70mM Bcl2
	geomean	26102.7	275167.5	19158.6	271464.2
			10.54172557		14.169313

B515LFC	control-LFC	17.5mM FITC	17.5mM BID	70mM FITC	70mM BID
	geomean	14595.4	118808.5	10294.1	86681.5
			8.140133193		8.420503007
AN2	aniridia-LFC	17.5mM FITC	17.5mM BID	70mM FITC	70mM BID
	geomean	16187.7	149072.8	13404.9	147158.2
			9.209016723		10.9779409
B550LFC	control-LFC	17.5mM FITC	17.5mM BID	70mM FITC	70mM BID
	geomean	10938.1	80207.1	12750.2	97740.6
			7.332818314		7.665809164
AN5	aniridia-LFC	17.5mM FITC	17.5mM BID	70mM FITC	70mM BID
	geomean	30416.9	287655.3	21914.4	171395.9
			9.457088		7.821154127
1748LFC	control-LFC	17.5mM FITC	17.5mM BID	70mM FITC	70mM BID
	geomean	20616.7	223202.8	13595.6	129894.9
			10.82631071		9.554186649
AN14	aniridia-LFC	17.5mM FITC	17.5mM BID	70mM FITC	70mM BID
	geomean	26102.7	215619.1	19158.6	193891.2
			8.260413674		10.12032194

B515LFC	control-LFC	17.5mM FITC	17.5mM caspase3	70mM FITC	70mM caspase3
	geomean	14595.4	129039.6	10294.1	47505.7
			8.841114324		4.61484734
AN2	aniridia-LFC	17.5mM FITC	17.5mM caspase3	70mM FITC	70mM caspase3
	geomean	16187.7	215342.5	13404.9	85035.9
			13.30284722		6.343642996
B550LFC	control-LFC	17.5mM FITC	17.5mM caspase3	70mM FITC	70mM caspase3
	geomean	10938.1	161029.1	12750.2	62923.8
			14.72185297		4.935122586
AN5	aniridia-LFC	17.5mM FITC	17.5mM caspase3	70mM FITC	70mM caspase3
	geomean	30416.9	384302.6	21914.4	130518.1
			12.63450911		5.955814442
1748LFC	control-LFC	17.5mM FITC	17.5mM caspase3	70mM FITC	70mM caspase3
	geomean	20616.7	160723.8	13595.6	85983.4
			7.795806312		6.324354938
AN14	aniridia-LFC	17.5mM FITC	17.5mM caspase3	70mM FITC	70mM caspase3
	geomean	26102.7	539728.4	19158.6	91218.6
			20.67711003		4.761235163

B515LFC	control-LFC	17.5mM FITC	17.5mM caspase7	70mM FITC	70mM caspase7
	geomean	14595.4	35594.6	10294.1	23756.3
			2.438754676		2.307758813
AN2	aniridia-LFC	17.5mM FITC	17.5mM caspase7	70mM FITC	70mM caspase7
	geomean	16187.7	56930.3	13404.9	35524.8
			3.516886278		2.650135398
B550LFC	control-LFC	17.5mM FITC	17.5mM caspase7	70mM FITC	70mM caspase7
	geomean	10938.1	45165.4	12750.2	36084.4
			4.129181485		2.830104626
AN5	aniridia-LFC	17.5mM FITC	17.5mM caspase7	70mM FITC	70mM caspase7
	geomean	30416.9	95202.7	21914.4	33568.5
			3.12992777		1.531801008
1748LFC	control-LFC	17.5mM FITC	17.5mM caspase7	70mM FITC	70mM caspase7
	geomean	20616.7	44892.6	13595.6	18680.7
			2.177487183		1.37402542
AN14	aniridia-LFC	17.5mM FITC	17.5mM caspase7	70mM FITC	70mM caspase7
	geomean	26102.7	54115.8	19158.6	27153.2
			2.073187831		1.417285188

B515LFC	control-LFC	17.5mM FITC	17.5mM caspase8	70mM FITC	70mM caspase8
	geomean	14595.4	37863.7	10294.1	14837.5
			2.594221467		1.441359614
AN2	aniridia-LFC	17.5mM FITC	17.5mM caspase8	70mM FITC	70mM caspase8
	geomean	16187.7	37996.5	13404.9	25489.5
			2.347245131		1.901506166
B550LFC	control-LFC	17.5mM FITC	17.5mM caspase8	70mM FITC	70mM caspase8
	geomean	10938.1	24291.2	12750.2	28357.9
			2.220787888		2.224114132
AN5	aniridia-LFC	17.5mM FITC	17.5mM caspase8	70mM FITC	70mM caspase8
	geomean	30416.9	95830.5	21914.4	27116.4
			3.150567612		1.237378162
1748LFC	control-LFC	17.5mM FITC	17.5mM caspase8	70mM FITC	70mM caspase8
	geomean	20616.7	43292.3	13595.6	29767.9
			2.099865643		2.189524552
AN14	aniridia-LFC	17.5mM FITC	17.5mM caspase8	70mM FITC	70mM caspase8
	geomean	26102.7	63301.1	19158.6	25436.2
			2.425078632		1.327664861

B515LFC	control-LFC	17.5mM FITC	17.5mM caspase9	70mM FITC	70mM caspase9
	geomean	14595.4	25254.6	10294.1	20128.9
			1.73031229		1.955382209
AN2	aniridia-LFC	17.5mM FITC	17.5mM caspase9	70mM FITC	70mM caspase9
	geomean	16187.7	39063.6	13404.9	40015.7
			2.413165552		2.985154682
B550LFC	control-LFC	17.5mM FITC	17.5mM caspase9	70mM FITC	70mM caspase9
	geomean	10938.1	14433.6	12750.2	21156.4
			1.319571041		1.659299462
AN5	aniridia-LFC	17.5mM FITC	17.5mM caspase9	70mM FITC	70mM caspase9
	geomean	30416.9	58104.6	21914.4	43818.7
			1.910273565		1.999539116
1748LFC	control-LFC	17.5mM FITC	17.5mM caspase9	70mM FITC	70mM caspase9
	geomean	20616.7	29593.6	13595.6	15854.8
			1.435418859		1.166171408
AN14	aniridia-LFC	17.5mM FITC	17.5mM caspase9	70mM FITC	70mM caspase9
	geomean	26102.7	31457.4	19158.6	30115.5
			1.205139698		1.571905045

B515LFC	control-LFC	17.5mM FITC	17.5mM caspase10	70mM FITC	70mM caspase10
	geomean	14595.4	240299.6	10294.1	173552.8
			16.46406402		16.85944376
AN2	aniridia-LFC	17.5mM FITC	17.5mM caspase10	70mM FITC	70mM caspase10
	geomean	16187.7	217947.5	13404.9	158415.2
			13.46377188		11.81770845
B550LFC	control-LFC	17.5mM FITC	17.5mM caspase10	70mM FITC	70mM caspase10
	geomean	10938.1	153512.9	12750.2	89672.6
			14.03469524		7.033034776
AN5	aniridia-LFC	17.5mM FITC	17.5mM caspase10	70mM FITC	70mM caspase10
	geomean	30416.9	551339.5	21914.4	236880.7
			18.12609109		10.80936279
1748LFC	control-LFC	17.5mM FITC	17.5mM caspase10	70mM FITC	70mM caspase10
	geomean	20616.7	228580.2	13595.6	106822.8
			11.0871381		7.857159669
AN14	aniridia-LFC	17.5mM FITC	17.5mM caspase10	70mM FITC	70mM caspase10
	geomean	26102.7	341804.4	19158.6	133644.7
			13.09459941		6.975702818

B515LFC	control-LFC	17.5mM FITC	17.5mM p21	70mM FITC	70mM p21
	Geometric mean	14595.4	45507.3	10294.1	32841.1
			3.117920715		3.190283755
AN2	aniridia-LFC	17.5mM FITC	17.5mM p21	70mM FITC	70mM p21
	geomean	16187.7	59294	13404.9	24070.1
			3.662904551		1.795619512
B550LFC	control-LFC	17.5mM FITC	17.5mM p21	70mM FITC	70mM p21
	geomean	10938.1	31767.4	12750.2	28862.2
			2.904288679		2.263666452
AN5	aniridia-LFC	17.5mM FITC	17.5mM p21	70mM FITC	70mM p21
	geomean	30416.9	96510.6	21914.4	44784.8
			3.172926893		2.043624284
1748LFC	control-LFC	17.5mM FITC	17.5mM p21	70mM FITC	70mM p21
	geomean	20616.7	51163.6	13595.6	29706.3
			2.481658073		2.184993674
AN14	aniridia-LFC	17.5mM FITC	17.5mM p21	70mM FITC	70mM p21
	geomean	26102.7	29078.6	19158.6	25415.4
			1.114007363		1.326579186

B515LFC	control-LFC	17.5mM FITC	17.5mM p27	70mM FITC	70mM p27
	geomean	7238.6	25518.6	9262.2	19468.4
			3.525350206		2.10191963
AN2	aniridia-LFC	17.5mM FITC	17.5mM p27	70mM FITC	70mM p27
	geomean	10587.6	37601.5	9661.1	28828
			3.551465866		2.983925226
B550LFC	control-LFC	17.5mM FITC	17.5mM p27	70mM FITC	70mM p27
	geomean	6920.5	28458.5	8317.4	30124.3
			4.112202876		3.62184096
AN5	aniridia-LFC	17.5mM FITC	17.5mM p27	70mM FITC	70mM p27
	geomean	11799.6	50950.2	9471.1	38910.9
			4.317959931		4.108382342
1748LFC	control-LFC	17.5mM FITC	17.5mM p27	70mM FITC	70mM p27
	geomean	8446.1	27843.4	11764.4	32170.1
			3.29659843		2.734529598
AN14	aniridia-LFC	17.5mM FITC	17.5mM p27	70mM FITC	70mM p27
	geomean	14701.9	39526.1	9143.2	30084.3
			2.688502847		3.290346924

B515LFC	control-LFC	17.5mM FITC	17.5mM Survivin	70mM FITC	70mM Survivin
	geomean	14595.4	89303.4	10294.1	229968.3
			6.118599011		22.33981601
AN2	aniridia-LFC	17.5mM FITC	17.5mM Survivin	70mM FITC	70mM Survivin
	geomean	16187.7	81081.9	13404.9	234243.1
			5.008858578		17.47443845
B550LFC	control-LFC	17.5mM FITC	17.5mM Survivin	70mM FITC	70mM Survivin
	geomean	10938.1	147304.4	12750.2	182093.6
			13.46709209		14.28162695
AN5	aniridia-LFC	17.5mM FITC	17.5mM Survivin	70mM FITC	70mM Survivin
	geomean	30416.9	217819.3	21914.4	257905.3
			7.161127531		11.76875935
1748LFC	control-LFC	17.5mM FITC	17.5mM Survivin	70mM FITC	70mM Survivin
	geomean	20616.7	193359.7	13595.6	205917.5
			9.37879001		15.14589279
AN14	aniridia-LFC	17.5mM FITC	17.5mM Survivin	70mM FITC	70mM Survivin
	geomean	26102.7	195572.4	19158.6	330225.8
			7.492420324		17.23642646

B515LFC	control-LFC	17.5mM FITC	17.5mM TNFα	70mM FITC	70mM TNFα
	geomean	14595.4	16883.8	10294.1	18431.3
			1.156789125		1.790472212
AN2	aniridia-LFC	17.5mM FITC	17.5mM TNFα	70mM FITC	70mM TNFα
	geomean	16187.7	32134.3	13404.9	20781.2
			1.985105976		1.550268932
B550LFC	control-LFC	17.5mM FITC	17.5mM TNFα	70mM FITC	70mM TNFα
	geomean	10938.1	17828.6	12750.2	16514.7
			1.629954014		1.295250271
AN5	aniridia-LFC	17.5mM FITC	17.5mM TNFα	70mM FITC	70mM TNFα
	geomean	30416.9	58436.6	21914.4	42087.6
			1.92118855		1.920545395
1748LFC	control-LFC	17.5mM FITC	17.5mM TNFα	70mM FITC	70mM TNFα
	geomean	20616.7	51216.1	13595.6	29556.8
			2.484204553		2.17399747
AN14	aniridia-LFC	17.5mM FITC	17.5mM TNFα	70mM FITC	70mM TNFα
	geomean	26102.7	39641.1	19158.6	32802.9
			1.518658989		1.712176255

B515LFC	control-LFC	17.5mM FITC	17.5mM XIAP	70mM FITC	70mM XIAP
	geomean	14595.4	92337.4	10294.1	137552.2
			6.326472724		13.36223662
AN2	aniridia-LFC	17.5mM FITC	17.5mM XIAP	70mM FITC	70mM XIAP
	geomean	16187.7	103443.1	13404.9	204741.3
			6.390228383		15.27361636
B550LFC	control-LFC	17.5mM FITC	17.5mM XIAP	70mM FITC	70mM XIAP
	geomean	10938.1	89443.6	12750.2	134416.7
			8.177251991		10.5423209
AN5	aniridia-LFC	17.5mM FITC	17.5mM XIAP	70mM FITC	70mM XIAP
	geomean	30416.9	89487.4	21914.4	201722.3
			2.942028938		9.205011317
1748LFC	control-LFC	17.5mM FITC	17.5mM XIAP	70mM FITC	70mM XIAP
	geomean	20616.7	91622.1	13595.6	175464.8
			4.444072039		12.905999
AN14	aniridia-LFC	17.5mM FITC	17.5mM XIAP	70mM FITC	70mM XIAP
	geomean	26102.7	78130.4	19158.6	202258.3
			2.993192275		10.5570501

apoptosis rate	LSCs			AN-LSCs		
17.5 mM	3.55	2.47	2.29	3.13	2.05	3.89
70 mM	0.51	0.37	1.02	0.58	1.17	0.98
